# Pterostilbene Attenuates Fructose-Induced Myocardial Fibrosis by Inhibiting ROS-Driven Pitx2c/miR-15b Pathway

**DOI:** 10.1155/2019/1243215

**Published:** 2019-12-04

**Authors:** Lin-Lin Kang, Dong-Mei Zhang, Rui-Qing Jiao, Shu-Man Pan, Xiao-Juan Zhao, Yan-Jing Zheng, Tian-Yu Chen, Ling-Dong Kong

**Affiliations:** ^1^State Key Laboratory of Pharmaceutical Biotechnology, School of Life Science, Nanjing University, Nanjing, China; ^2^State Key Laboratory Cultivation Base for TCM Quality and Efficacy, Nanjing University of Chinese Medicine, Nanjing 210023, China

## Abstract

Excessive fructose consumption induces oxidative stress and myocardial fibrosis. Antioxidant compound pterostilbene has cardioprotective effect in experimental animals. This study is aimed at investigating how fructose drove fibrotic responses via oxidative stress in cardiomyocytes and explored the attenuation mechanisms of pterostilbene. We observed fructose-induced myocardial hypertrophy and fibrosis with ROS overproduction in rats. Paired-like homeodomain 2 (Pitx2c) increase, microRNA-15b (miR-15b) low expression, and p53 phosphorylation (p-p53) upregulation, as well as activation of transforming growth factor-*β*1 (TGF-*β*1)/drosophila mothers against DPP homolog (Smads) signaling and connective tissue growth factor (CTGF) induction, were also detected in fructose-fed rat hearts and fructose-exposed rat myocardial cell line H9c2 cells. The results from *p53* siRNA or *TGF-β1* siRNA transfection showed that TGF-*β*1-induced upregulation of CTGF expression and p-p53 activated TGF-*β*1/Smads signaling in fructose-exposed H9c2 cells. Of note, Pitx2c negatively modulated miR-15b expression *via* binding to the upstream of the miR-15b genetic loci by chromatin immunoprecipitation and transfection analysis with pEX1-Pitx2c plasmid and *Pitx2c* siRNA, respectively. In H9c2 cells pretreated with ROS scavenger N-acetylcysteine, or transfected with miR-15b mimic and inhibitor, fructose-induced cardiac ROS overload could drive Pitx2c-mediated miR-15b low expression, then cause p-p53-activated TGF-*β*1/Smads signaling and CTGF induction in myocardial fibrosis. We also found that pterostilbene significantly improved myocardial hypertrophy and fibrosis in fructose-fed rats and fructose-exposed H9c2 cells. Pterostilbene reduced cardiac ROS to block Pitx2c-mediated miR-15b low expression and p-p53-dependent TGF-*β*1/Smads signaling activation and CTGF induction in high fructose-induced myocardial fibrosis. These results firstly demonstrated that the ROS-driven Pitx2c/miR-15b pathway was required for p-p53-dependent TGF-*β*1/Smads signaling activation in fructose-induced myocardial fibrosis. Pterostilbene protected against high fructose-induced myocardial fibrosis through the inhibition of Pitx2c/miR-15b pathway to suppress p-p53-activated TGF-*β*1/Smads signaling, warranting the consideration of Pitx2c/miR-15b pathway as a therapeutic target in myocardial fibrosis.

## 1. Introduction

Fructose overconsumption increases oxidative stress, inflammation, and cardiomyocyte hypertrophy, causing myocardial fibrosis [1, 2]. Transforming growth factor-*β*1/(small) mothers against decapentaplegic homologs (TGF-*β*1/Smads) signaling is known to mediate the pathological process of fibrosis. Its activation is observed in left ventricle tissues of Western diet-fed mice with myocardial fibrosis [3]. TGF-*β*1 activates the promoter of connective tissue growth factor (CTGF) to induce its expression in rat primary cardiac myocytes, in parallel with myocardial infarction in rats and cardiac ischemia patients [4]. Activation of Smad3/4 is essential for TGF-*β*1-induced CTGF transcription in rat proximal tubular epithelial cells with the progression of tubulointerstitial fibrosis [5]. Consistently, high levels of hyaluronic acid, hydroxyproline, and collagen volume fraction (important indicators in clinical diagnosis of myocardial fibrosis) are observed in heart tissues from hypertrophic cardiomyopathy patients [6–8]. Furthermore, mRNA expression levels of cardiac hypertrophy-related genes such as atrial natriuretic peptide (ANP), brain natriuretic peptide (BNP), and beta myosin heavy chain (*β*-MHC) and fibrosis-related genes such as collagen I, collagen III, CTGF, and TGF-*β*1 are also increased in aortic banding-induced experimental cardiac hypertrophy and fibrosis [9]. Of note, TGF-*β*1, alpha smooth muscle-actin (*α*-SMA), and fibroblast specific-1 (FSP-1) are increased in mouse hearts and the primary cardiomyocytes during fructose-induced myocardial fibrosis [10]. How high fructose intake causes myocardial fibrosis and its possible pathological mechanism are still unknown.

Recent study shows that the microRNA-15 (miR-15) family acts as a novel regulator of cardiac hypertrophy and fibrosis by inhibiting TGF-*β* pathway [11]. Early downregulation of miR-15b precedes the activation of profibrogenic mediators and then accelerates fibrotic remodeling in the hearts of type-2 diabetic mice [12]. Moreover, a bioinformatics approach predicts that osteoblastic specific miR-15b targets 16 genes in the tumor suppressor p53 signaling pathway [13]. Interestingly, p53 as a highly labile transcription factor is increased in myocardial biopsies of patients with heart disease [14]. Heart overexpression of p53 and TGF-*β*1 is also detected in high oxygen-exposed rats with cardiomyocyte hypertrophy and enhanced fibrosis [15]. A specific p53 inducer, indoxyl sulfate, can enhance p53-TGF-*β*1/Smad3 pathway in kidney fibrosis of rats [16]. However, it is unclear whether miR-15b regulates p-p53 to activate TGF-*β*1/Smads signaling in fructose-induced myocardial fibrosis.

Oxidative stress and reactive oxygen species (ROS) overproduction cause myocardial damage during the progression of myocardial fibrosis [17]. p53 acts as a finely tuned regulator of redox-dependent physiological processes [18]. Redox regulation by paired-like homeodomain transcription factor 2 (Pitx2) affects cardiac structure and function [19, 20]. Pitx2 promotes heart repair by activating antioxidant response after cardiac injury [20]. Surprisingly, Pitx2c is reported to negatively regulate miR-15b expression in cell proliferation of myoblasts [21]. In fact, fructose induces oxidative stress in myocardial fibrosis of rats [17]. The possible molecular mechanism by which fructose affects Pitx2c via oxidative stress to dysregulate miR-15b in myocardial fibrosis needs to be explored.

Pterostilbene, a natural dimethylated resveratrol analog mainly from blueberries and grape vines [22], shows pleiotropic pharmacological actions, including antioxidation and anti-inflammation [23]. Pterostilbene significantly inhibits creatine kinase (CK) and creatine kinase isoenzyme (CK-MB) activities in serum and reduces nicotinamide adenine dinucleotide phosphate (NADPH) oxidase-dependent oxidative stress, showing its cardioprotective effect on myocardial ischemia/reperfusion injury, right ventricle hypertrophy, and contractile dysfunction in monocrotaline-induced pulmonary hypertension of rats [24–26]. It also reduces oxidative stress and p53 overexpression in oxidized low-density lipoprotein-induced human umbilical vein endothelial cell apoptosis [27], inhibits TGF-*β*1/Smads signaling and *α*-SMA expression, and alleviates dimethylnitrosamine-induced liver fibrosis in rats [28]. These observations indicate that pterostilbene may relieve myocardial fibrosis under fructose-induced oxidative stress.

In this study, we investigated whether fructose induced Pitx2c to negatively regulate miR-15b in myocardial fibrosis and examined what the molecular basis could be. Our findings demonstrated that fructose-induced ROS overload increased Pitx2c to downregulate miR-15b expression, then activated p-p53-dependent TGF-*β*1/Smads signaling, causing CTGF-mediated myocardial fibrosis. Pterostilbene with antioxidation downregulated Pitx2c to upregulate miR-15b and reduced p-p53 to suppress TGF-*β*1/Smads signaling activation and CTGF expression in the attenuation of fructose-induced myocardial fibrosis.

## 2. Materials and Methods

### 2.1. Animals and Treatments

Male Sprague-Dawley rats (6-7 weeks, 180-220 g) were obtained from the Experimental Animal Centre of Zhejiang Province (Hangzhou, China) (Production license: SCXK 2014-0001). All animals were put on a 22 ± 2°C (humidity of 55 ± 5%) housing condition with controlled 12 h light/dark cycle (lights on from 6:30 a.m.-6:30 p.m.) and fed with standard laboratory chow and water *ad libitum* throughout the experiments. 10% (wt/vol) fructose (Shandong Xiwang Sugar Industry Co., Ltd., Binzhou, China) was offered in water with standard chow for 6 weeks. Then, these animals were divided indiscriminately into 5 subgroups (*n* = 8/group), orally receiving (3:00-4:00 p.m.) drinking water; 10, 20, and 40 mg/kg pterostilbene (97% purity); and 5 mg/kg allopurinol (98% purity) (Sigma, St. Louis, MO, USA) for another 11 weeks.

Pterostilbene (25, 50, and 100 mg/kg) is reported to ameliorate cardiac oxidative stress, hypertrophy, and right ventricle systolic dysfunction in monocrotaline-induced pulmonary hypertension of rats [26]. It also alleviates myocardial ischemia/reperfusion injury of rats at 10 mg/kg [24]. Allopurinol, with antioxidative activity, is clinically used to treat some cardiovascular diseases [29, 30]. Our previous studies showed that pterostilbene (10, 20, and 40 mg/kg) or allopurinol (5 mg/kg) reduced fructose-induced oxidative stress and inflammation in the heart, liver, or kidney of fructose-fed rats [17, 31–33]. Additionally, allopurinol restores a high-fat and high-fructose diet-induced myocardial oxidative stress, cardiomyocyte hypertrophy, interstitial fibrosis, and left ventricular diastolic dysfunction in mice [34] as well as ventricular relaxation impairment and cardiac ischemia in rats [35]. Thus, 10, 20, and 40 mg/kg were selected as the dosages of pterostilbene administration, and 5 mg/kg allopurinol was the positive control in this study. Rat body weight was measured weekly. Animal welfare and experimental procedures were carried out in accordance with the recommendations in the *Guide for the Care and Use of Laboratory Animals* prepared by the National Academy of Sciences and published by the National Institutes of Health (NIH publication 86-23 revised 1985).

### 2.2. Collection of Blood and Tissue Samples

During the last feeding week, animals were anesthetized according to a previously described protocol [17]. Serum samples were collected by centrifugation (4000 × *g*, 4°C) for 10 min and stored at -80°C for biochemical assays. Heart tissue samples were rapidly dissected on ice and stored at -80°C for microarray, biochemical, qRT-PCR, and Western blot assay, respectively, while some of which were fixed for histological study.

### 2.3. miRNA Microarray Analysis

A microarray-based approach was used to identify miRNA expression difference in plasma samples between normal and fructose-fed rats [36]. The microarray analysis for miRNA profiling using the miRCURY LNA Array system (Exiqon, Vedbaek, Denmark) was conducted by the KangChen Bio-tech Inc. (Shanghai, China). The threshold value for significance used to define upregulation or downregulation of miRNAs was a fold change > 1.5 or <0.6. Here, circulating levels of miR-15b showed a relatively obvious downtrend with fold change of 0.5641 (fructose vehicle *vs*. normal control *P* value = 0.0505) ([Supplementary-material supplementary-material-1]).

### 2.4. Determination of Serum CK-MB, Troponin (cTn-T), CK, and Myoglobin (MB) Levels

CK-MB, cTn-T, CK, and MB levels in serum were analyzed with standard diagnostic kits (Jiancheng Biotechnology Co., Ltd., Nanjing, China), respectively.

### 2.5. Immunohistochemistry Analysis

Rat heart tissues were stained with hematoxylin-eosin (HE) or Masson trichrome staining, according to our previously described protocols, respectively [17, 32].

### 2.6. Determination of Hydroxyproline and Hyaluronic Acid Levels in Rat Hearts

Rat heart tissues were homogenized in sodium chloride (10 wt/vol) on ice and then centrifuged (10000 × *g*, 4°C) for 15 min to obtain the supernatants. Hydroxyproline and hyaluronic acid levels were measured by standard diagnostic kits (Jiancheng Biotechnology Co., Ltd., Nanjing, China), respectively.

### 2.7. Fluorescence *In Situ* Hybridization (FISH) for miR-15b Detection in Rat Hearts

miR-15b-FISH detection was performed according to a previously described protocol with some modifications as listed below [11]. miR-15b probe was synthesized (Wuhan Servicebio Technology Co., Ltd., Wuhan, China), and then, the FISH Tag RNA Green Kit with Alexa Fluor 488 was used (Invitrogen, Burlington, ON, Canada). The sequence of Rat-miR-15b probe for *in situ* hybridization was 5′-TGTAA ACCAT GATGTGCTGC TA-3′.

Nuclear and cytoplasmic staining for detecting miRNA precursors and mature miRNA was carried out in cardiomyocytes, the images of which were obtained using an upright microscope (Nikon ECLIPSE CI, Nikon, Japan).

### 2.8. Cell Culture and Treatment

Rat myocardial cell line H9c2 cells were obtained by Shanghai Fuxiang Biotechnology Co., Ltd. (Shanghai, China) and cultured according to our previously described protocol [17]. These cells were cultivated in DMEM (containing 10% FBS) and exposed to 0.1% DMSO (normal control); 5 mM fructose (fructose-vehicle); 5 mM fructose coincubated with 2.5, 5, and 10 *μ*M pterostilbene; or 30 *μ*M allopurinol for 24 or 48 h, respectively.

H9c2 cells were incubated in serum-free DMEM for 12 h. These cells were pretreated with ROS scavenger N-acetylcysteine (NAC, 1 mM, Amresco, Solon, USA) for 1 h and then coincubated with 5 mM fructose in the presence or absence of pterostilbene (10 *μ*M) or allopurinol (30 *μ*M) for further 24 or 48 h.

*p53* siRNA, miR-15b mimic, miR-15b inhibitor, *CTGF* siRNA, or *TGF-β1* siRNA, as well as the respective negative control, were synthesized by GenePharma (Shanghai, China), respectively. These RNA sequences were listed in [Supplementary-material supplementary-material-1]. *p53* siRNA, miR-15b mimic, miR-15b inhibitor, *CTGF* siRNA, or *TGF-β1* siRNA (50 nM), as well as the negative controls in H9c2 cells, were incubated with Lipofectamine 2000 (Invitrogen, Carlsbad, CA, USA) for 6 h, respectively. The efficiency of the transfection was detected by qRT-PCR (Figures [Supplementary-material supplementary-material-1]–[Supplementary-material supplementary-material-1]). After the transfection, these cells were coincubated with 5 mM fructose in the presence or absence of pterostilbene (10 *μ*M) or allopurinol (30 *μ*M) for another 24 or 48 h.

H9c2 cells were transfected with pEX1-Pitx2c plasmid (1 *μ*g/mL) or *Pitx2c* siRNA (50 nM) (GenePharma) for 6 h and then used to determine miR-15b expression for another 24 h. These sequences were listed in Tables [Supplementary-material supplementary-material-1]. The transfection efficiency (Figures [Supplementary-material supplementary-material-1]) in H9c2 cells was detected by qRT-PCR, respectively.

Pterostilbene at 3 *μ*M is reported to attenuate hypoxia/reoxygenation-induced H9c2 cell injury [37]. 1 *μ*M pterostilbene reduces fructose-induced podocyte oxidative stress and inflammation [31]. And 5-20 *μ*M pterostilbene protects primary human corneal epithelial cells from hyperosmotic stress-induced inflammatory injury and oxidative stress [38]. Pterostilbene (1.25, 2.5, or 5 *μ*M) attenuates cerebral ischemia reperfusion-induced mitochondrial oxidative damage [39]. In our previous studies [17, 31, 33], allopurinol restored fructose-induced ROS overproduction in H9c2 cells at 30 *μ*M, primary rat hepatocytes at 5 *μ*M, or mouse podocytes at 100 *μ*M. Accordingly, pterostilbene at 2.5, 5, and 10 *μ*M, as well as allopurinol at 30 *μ*M, was selected for the cell experiments.

Pterostilbene and allopurinol were dissolved in DMSO, while NAC was dissolved in ultrapure water at the respective stock concentrations. The concentrations of DMSO in all cell cultures were less than 0.1%. Cell culture supernatants and lysates were collected separately. Total RNAs or proteins were extracted and stored at -80°C for further biochemical, qRT-PCR, or Western blot analysis, respectively.

### 2.9. Determination of Oxidative Stress

Determination of oxidative stress was performed according to the previously described protocol [17, 40]. Total ROS levels were measured using a commercial kit (Beyotime Institute of Biotechnology, Haimei, China). Activity of NADPH oxidase was represented as the rate of NADPH consumption as previously described [40]. Malondialdehyde (MDA) levels were determined by a standard diagnostic kit (Jiancheng Biotechnology Co., Ltd., Nanjing, China).

### 2.10. Immunofluorescence (IF) Analysis

Heart samples were snap frozen in prechilled isopentane (fill a glass beaker with isopentane and keep on dry ice) and stored at -80°C. The frozen samples were cut into 8 *μ*m thick sections. The IF assay for rat heart tissue and H9c2 cell samples was performed according to the previously described protocols with minor modifications [41]. Briefly, except ANP used paraffin-embedded sections of the heart herein, frozen sections of the heart and cells were fixed using 4% formaldehyde, then incubated overnight at 4°C with a polyclonal anti-rabbit ANP (ab76743, dilution 1 : 200), a polyclonal anti-rabbit *α*-SMA (ab5694, dilution 1 : 200), a monoclonal anti-mouse SPG3A (namely, FSP-1, ab58273, dilution 1 : 100), a polyclonal anti-rabbit CTGF (ab125943, dilution 1 : 200) (Abcam, Cambridge, MA, USA), and a polyclonal anti-rabbit TGF-*β*1 (sc-146, 1 : 500; Santa Cruz, CA, USA), respectively. The secondary antibodies conjugated to an Alexa Fluor 555 goat anti-rabbit IgG, 555 goat anti-mouse IgG, and 488 goat anti-rabbit IgG (A-21428; A-21424; A-11008; Life Technologies, Oregon, USA) were added and incubated for 0.5-1 h further at room temperature. After rinsing, the slides were incubated with DAPI for 8 min for nucleus staining. Images were acquired by a Multiphoton Confocal Microscope (Lei TCS SP8-MaiTai MP, State Key Laboratory of Pharmaceutical Biotechnology, Nanjing University, Nanjing, China) and processed with Photoshop software (Adobe, San Jose, CA, USA).

### 2.11. Chromatin Immunoprecipitation (ChIP) Assay

ChIP assay was performed as previously described using an EpiQuik™ ChIP Kit (EpiGentek, New York, USA) with the following modifications [42]. H9c2 cells were transfected with 10 *μ*g pEX1-Pitx2c plasmid using Lipofectamine 2000 in a 75 cm^2^ culture flask. After 6 h, cells were swapped by Opti-MEM® I Reduced-Serum Medium (Invitrogen) for another 24 h and then cross-linked with 1% formaldehyde for 10 min at room temperature on a rocking platform, lysed, and sonicated. 1% of the sheared DNA-protein complex was used for an input DNA sample. Soluble chromatin was incubated for 90 min at room temperature with anti-Pitx2 (ab192495; Abcam, MA, USA) and anti-RNA polymerase II as a positive control or normal mouse IgG as a negative control (supplied by the kit), respectively. All PCRs were performed at an annealing temperature of 55°C. Different primers were used to amplify the DNA regions containing the Pitx2c binding site 6 kb upstream of the coding sequences for miR-15b ([Supplementary-material supplementary-material-1]). Normal mouse IgG exhibited nonspecific immunoprecipitation with chromatin. Glyceraldehyde-3-phosphate dehydrogenase (GAPDH) used as a positive control showed the efficacy of the kit reagents and protocols ([Supplementary-material supplementary-material-1]); its primers were illustrated in [Supplementary-material supplementary-material-1]. Three parallel qRT-PCRs were performed in triplicate with dilutions of input DNA to determine the linear range of amplification.

### 2.12. RNA Isolation and qRT-PCR Analysis

Total RNA was isolated from rat heart tissues and H9c2 cells using TRIzol reagent (Invitrogen). The primers were synthesized by Shanghai Generay Biotech Co., Ltd. (Shanghai, China), and the sequences were listed in [Supplementary-material supplementary-material-1].

For mRNA qRT-PCR analysis, single-stranded cDNA was reverse transcribed from RNA using oligo-dT primers. For miRNA qRT-PCR analysis, miRNA was extracted with specific stem loop from RNA by reverse transcription. Reverse transcription was conducted using a HiScript® II Q RT SuperMix (Vazyme, Nanjing, China) and iQ™ SYBR® Green Supermix (Bio-Rad Laboratories, Hercules, CA, USA), respectively.

The qRT-PCR analysis was performed according to our previously described protocol [17]. The relative expression of mRNAs was normalized to GAPDH, while that of miRNAs was normalized to U6, respectively.

### 2.13. Western Blot Analysis

Western blot analysis was performed according to the previous report [17]. Rat heart tissue samples were homogenized in lysis buffer, and cell lysates were centrifuged at 12000 × *g* for 20 min at 4°C. The primary antibodies in Western blot analysis included the following: rabbit anti-p53 (#9282) and rabbit anti-Phospho-p53 (#9284) (dilution 1 : 1000) purchased from Cell Signaling Technology (Cambridge, USA); rabbit anti-Pitx2 (ab192495), rabbit anti-ANP (ab76743), rabbit anti-*α*-SMA (ab5694), rabbit anti-S100A4 (also named FSP-1) (ab197896), and rabbit anti-CTGF (ab125943) (dilution 1 : 1000) purchased from Abcam (Cambridge, MA, USA); goat anti-Smad2 (sc-6200), rabbit anti-Phospho-Smad2 (sc-101801), mouse anti-Smad3 (sc-101154), rabbit anti-Phospho-Smad3 (sc-130218), mouse anti-Smad4 (sc-7966), rabbit anti-GAPDH (sc-25778), and rabbit anti-TGF-*β*1 (sc-146) (dilution 1 : 1000) purchased from Santa Cruz Biotechnology Co., Ltd. (CA, USA); and rabbit anti-*β*-actin (#SAP1647, dilution 1 : 12000) obtained from Sunshine Bio-Tech Co., Ltd. (Nanjing, China). After incubating with primary antibodies, blots were hatched with HRP-conjugated anti-rabbit IgG antibody (074-1506, dilution 1 : 10000, KPL) or HRP-conjugated anti-mouse IgG antibody (sc-2005, dilution 1 : 10000, Santa Cruz Biotechnology Co., Ltd.). Immunoreactive bands were visualized via enhanced chemiluminescence (Cell Signaling Technology) and quantified using ImageJ software (version 1.48v, NIH, Bethesda, MD).

### 2.14. Statistical Analysis

All data were analyzed with one-way analysis of variance (ANOVA) and followed by Tukey's Multiple Comparisons Test. Data were presented as the mean ± S.E.M., and *P* < 0.05 was considered as statistically significant.

## 3. Results

### 3.1. Pterostilbene Alleviates Fructose-Induced Myocardial Injury in Rats

**Figure 1:**
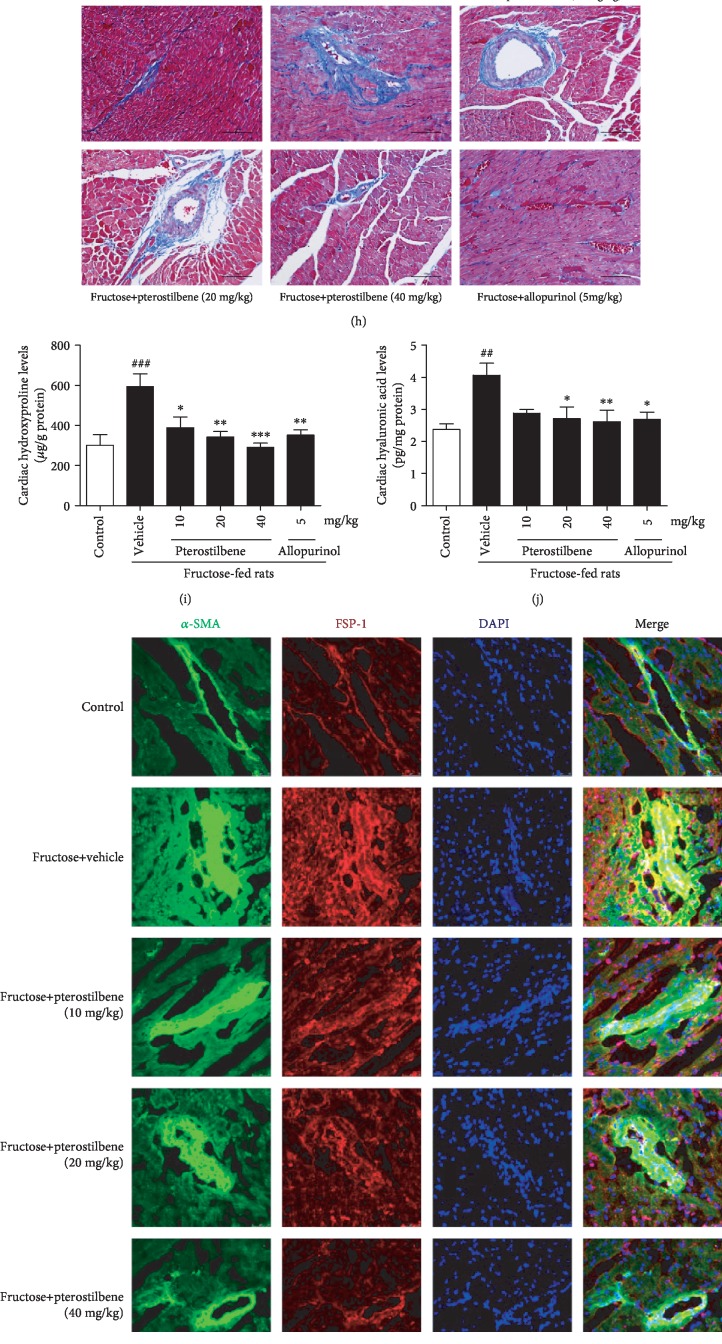
Pterostilbene and allopurinol improve myocardial injury in fructose-fed rats. Serum levels of CK-MB (a), cTn-T (b), CK (c), and MB (d) were measured, respectively (*n* = 8). HW/BW (e) was measured (*n* = 8). Histology of paraffin-embedded heart sections in different groups stained with ANP ((f), red) by immunofluorescence analysis (scale bar 75 *μ*m), HE ((g), scale bar 100 *μ*m), and Masson trichrome ((h), scale bar 100 *μ*m) by immunohistochemistry analysis, respectively. Heart levels of hydroxyproline (i) and hyaluronic acid (j) were measured (*n* = 8). Histology of frozen heart sections stained with *α*-SMA (green) and FSP-1 (red) ((k), scale bar 50 *μ*m), TGF-*β*1 ((l), red, scale bar 50 *μ*m), and CTGF ((m), red, scale bar 75 *μ*m) by immunofluorescence analysis, respectively. Herein, DAPI was used for staining nuclei, and merged views are shown in the right panels. Data are expressed as the mean ± S.E.M.^##^*P* < 0.01, ^###^*P* < 0.001*vs.* normal animal control group; ^∗^*P* < 0.05, ^∗∗^*P* < 0.01, and ^∗∗∗^*P* < 0.001*vs.* fructose-vehicle animal group.

As expected [17], serum levels of cardiac injury biomarkers CK-MB, cTn-T, CK, and MB were significantly raised in fructose-fed rats (Figures 1(a)–1(d)), which were restored by pterostilbene at a dose-dependent manner (Figures 1(a)–1(d)). Allopurinol had similar effects in this animal model (Figures 1(a)–1(d)). These data indicate that pterostilbene and allopurinol prevent fructose-induced myocardial injury in rats.

### 3.2. Pterostilbene Reduces Fructose-Induced Cardiomyocyte Hypertrophy and Fibrosis

**Figure 2:**
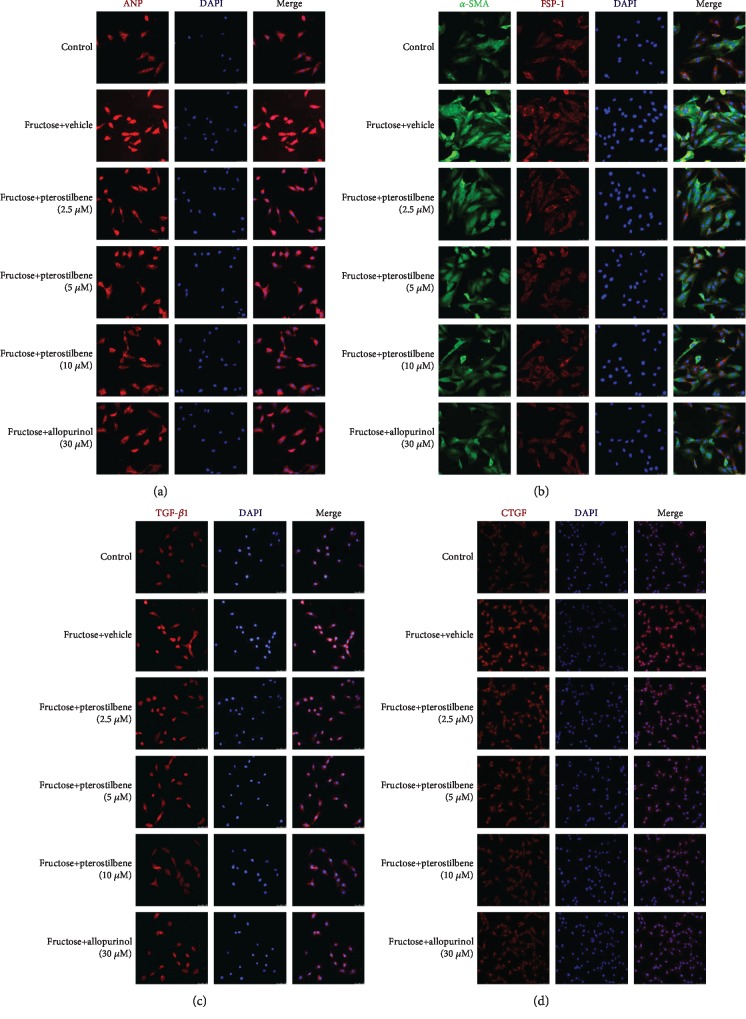
Pterostilbene and allopurinol reduce cellular fibrotic response-associated indicators ANP, *α*-SMA, FSP-1, TGF-*β*1, and CTGF expressions in fructose-exposed H9c2 cells. H9c2 cells in different groups stained with ANP ((a), red, scale bar 50 *μ*m), *α*-SMA (green) and FSP-1 (red) ((b), scale bar 50 *μ*m), TGF-*β*1 ((c), red, scale bar 50 *μ*m), and CTGF ((d), red, scale bar 75 *μ*m) by immunofluorescence analysis, respectively. DAPI was used for staining nuclei, and merged views were shown in the right panels.

Heart-to-body weight (HW/BW) was increased significantly in fructose-fed rats (Figure 1(e)). Expression levels of heart hypertrophic marker ANP were strongly increased in fructose-fed rats (Figure 1(f)). Consistently, morphological abnormality, including focal cell necrosis, disorganized array of myocardial structure, and myofibrillar discontinuation were detected (Figure 1(g)). Meanwhile, Masson trichrome staining showed significant collagen accumulation in both perivascular and interstitial tissues, while bundles of myofibers packed less tightly and separated by thick layers of fibrous tissues (Figure 1(h)), being consistent with high levels of hydroxyproline and hyaluronic acid in fructose-fed rat hearts (Figures 1(i) and 1(j)). High expression of cardiac *α*-SMA, FSP-1, TGF-*β*1, and CTGF was observed by IF assay along fibrotic septa (Figures 1(k)–1(m)). Furthermore, significant upregulation of ANP, *α*-SMA, FSP-1, TGF-*β*1, and CTGF was also detected in H9c2 cells incubated with 5 mM fructose (Figures 2(a)–2(d)).

Pterostilbene and allopurinol alleviated fructose-induced heart pathology in rats (Figures 1(f)–1(h)). Simultaneously, they downregulated cardiac *α*-SMA, FSP-1, TGF-*β*1, and CTGF expression (Figures 1(k)–1(m)) as well as hydroxyproline and hyaluronic acid levels (Figures 1(i) and 1(j)) in fructose-fed rats. Similar results were also confirmed in fructose-exposed H9c2 cells treated with pterostilbene and allopurinol, respectively (Figures 2(a)–2(d)), indicating that pterostilbene and allopurinol ameliorate fructose-induced myocardial hypertrophy and fibrosis.

### 3.3. Pterostilbene Downregulates p-p53 Phosphorylation to Inhibit TGF-*β*1/Smads Signaling Activation in Fructose-Exposed Cardiomyocytes

**Figure 3:**
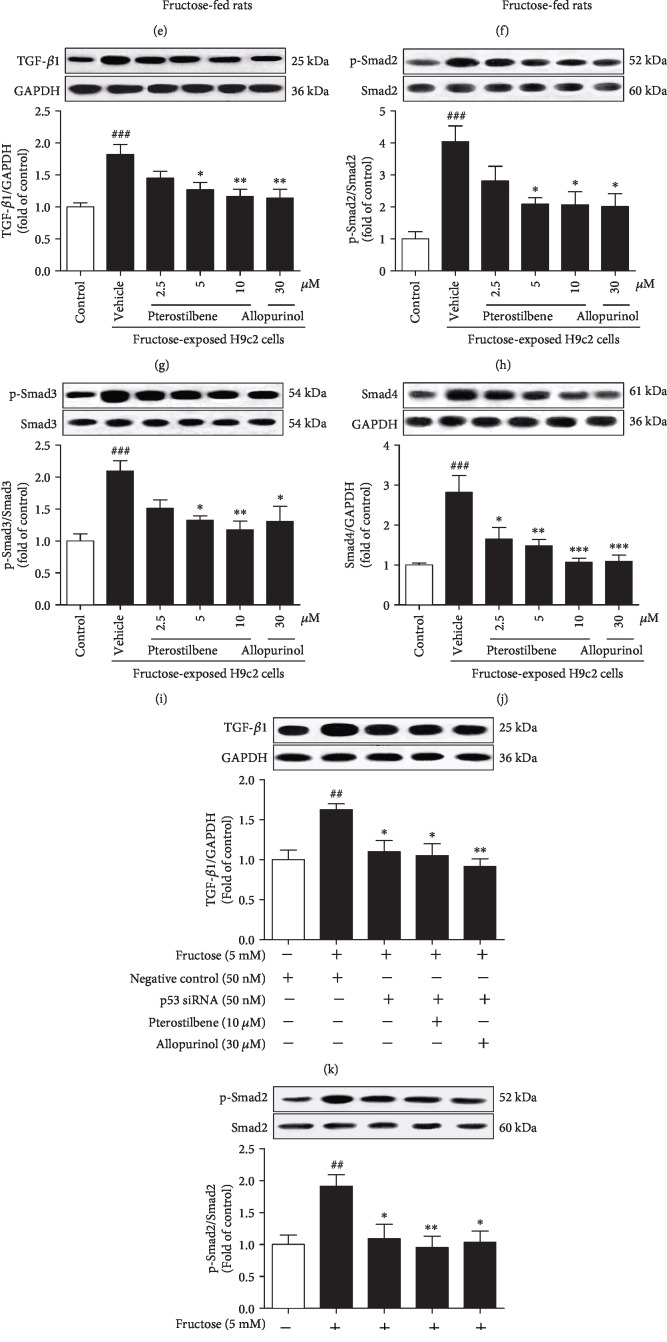
Pterostilbene and allopurinol downregulate p-p53 to inhibit TGF-*β*1/Smads signaling activation in fructose-fed rat hearts and fructose-exposed H9c2 cells. Protein levels of p-p53 were measured in fructose-fed rat hearts (a) and fructose-exposed H9c2 cells (b) (*n* = 6), respectively. Protein levels of TGF-*β*1, p-Smad2/3, and Smad4 were measured in fructose-fed rat hearts (c–f) and fructose-exposed H9c2 cells (g–j) (*n* = 6), respectively. Cellular protein levels of TGF-*β*1 (k), p-Smad2/3 (l, m), and Smad4 (n) were determined in *p53* siRNA-transfected H9c2 cells coincubated with 5 mM fructose, 10 *μ*M pterostilbene, and 30 *μ*M allopurinol (*n* = 6), respectively. Relative protein levels of TGF-*β*1 and Smad4 were normalized to GAPDH and of p-Smad2/3 were normalized to Smad2/3, respectively. Data are expressed as the mean ± S.E.M.^##^*P* < 0.01, ^###^*P* < 0.001*vs.* normal animal control group or normal cell control group; ^∗^*P* < 0.05, ^∗∗^*P* < 0.01, and ^∗∗∗^*P* < 0.001*vs.* fructose-vehicle animal group or fructose-vehicle cell group or fructose-vehicle+*p53* siRNA control cell group.

Protein levels of p53 phosphorylation (p-p53^ser15^) but not p53 were significantly upregulated in rat hearts (Figure 3(a)) and H9c2 cells (Figure 3(b)) under fructose exposure. Consistently, TGF-*β*1, p-Smad2/3, and Smad4 protein levels were increased in these animal and cell models (Figures 3(c)–3(j)). We further found that *p53* siRNA blocked fructose-induced change of TGF-*β*1, p-Smad2/3, and Smad4 in H9c2 cells (Figures 3(k)–3(n)).

Pterostilbene and allopurinol significantly downregulated cardiac p-p53 protein levels (Figures 3(a) and 3(b)) and inhibited TGF-*β*1/Smads signaling activation in fructose-fed rats and fructose-exposed H9c2 cells (Figures 3(c)–3(j)). More crucially, in *p53* siRNA-transfected H9c2 cells, pterostilbene and allopurinol had no effect on fructose-induced changes of cellular TGF-*β*1, p-Smad2/3, and Smad4 (Figures 3(k)–3(n)).

### 3.4. Pterostilbene Increases miR-15b Expression to Suppress p-p53-Dependent TGF-*β*1/Smads Signaling Activation in Cardiomyocytes under Fructose Induction

**Figure 4:**
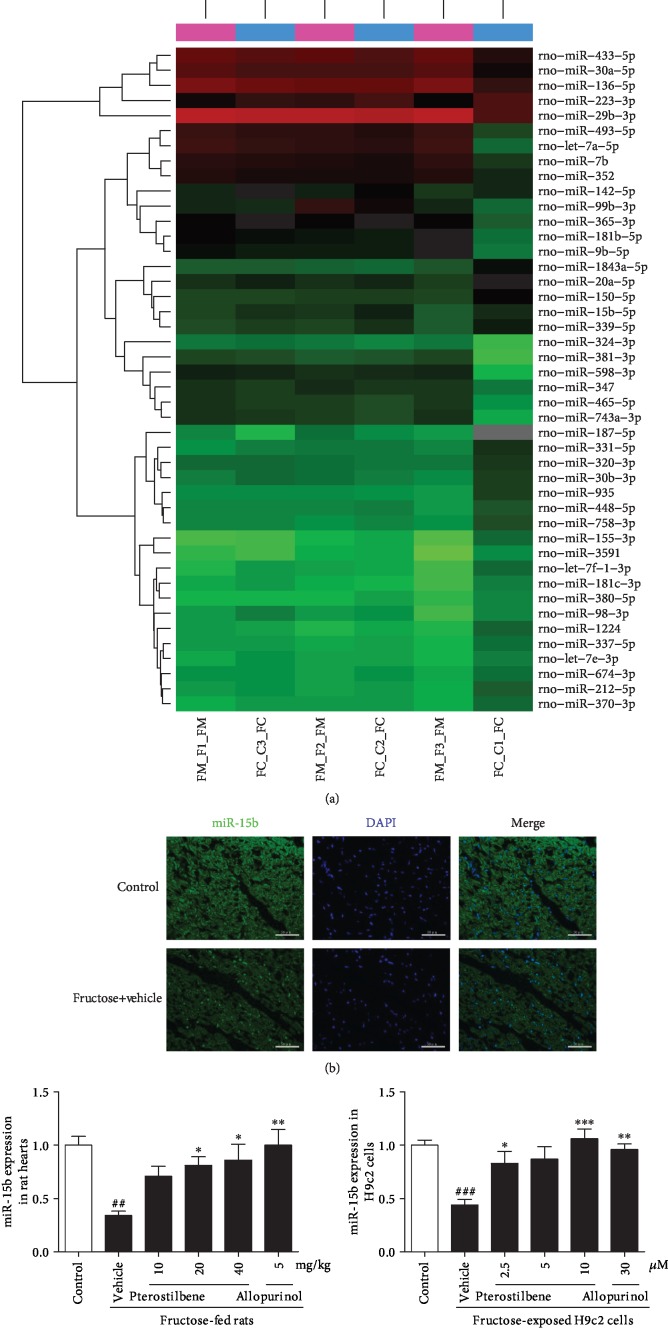
Pterostilbene and allopurinol restore fructose-induced miR-15b low expression in rat hearts and H9c2 cells. Cluster analysis of aberrant miRNA expression in plasma samples of fructose-fed rats according to a microarray scan (a); dendrogram generated by cluster analysis showing the separation of FM (normal animal control group) from FC (fructose-vehicle animal group) samples based on miRNA profiling (*n* = 3) (red: upregulation; green: downregulation). *In situ* hybridization showed miR-15b downregulation ((b), green) in the heart of fructose-fed rats (scale bar 50 *μ*m). DAPI was used for staining nuclei, and merged views were shown in the right panels. miR-15b expression was measured in rat hearts (c) and H9c2 cells (d) by qRT-PCR analysis (*n* = 6). The relative miR-15b expression levels were normalized to U6. Data are expressed as the mean ± S.E.M.^##^*P* < 0.01, ^###^*P* < 0.001*vs.* normal animal control group or normal cell control group; ^∗^*P* < 0.05, ^∗∗^*P* < 0.01, and ^∗∗∗^*P* < 0.001*vs.* fructose-vehicle animal group or fructose-vehicle cell group.

Microarray analysis showed an obvious downtrend of plasma miR-15b content in fructose-fed rats (Figure 4(a), [Supplementary-material supplementary-material-1]). We performed *in situ* hybridization and showed that fructose feeding significantly decreased rat heart miR-15b expression (Figure 4(b)), which was further confirmed in rat hearts and H9c2 cells under fructose condition (Figures 4(c) and 4(d)), indicating that miR-15b may be a potential noninvasive biomarker in fructose-induced rat heart fibrosis.

**Figure 5:**
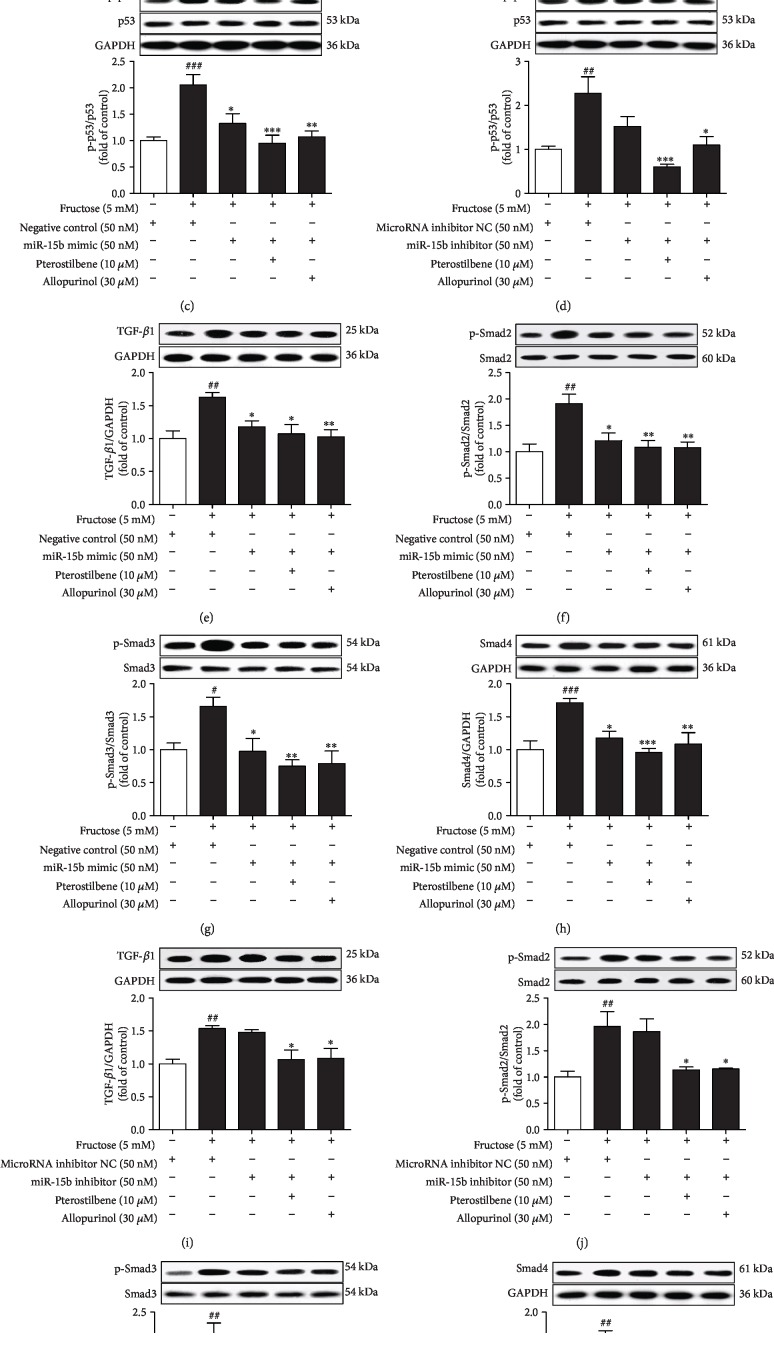
Pterostilbene and allopurinol increase miR-15b expression to suppress p-p53-dependent TGF-*β*1/Smads signaling activation in fructose-exposed H9c2 cells. Cellular levels of p-p53 protein (a) and miR-15b expression (b) were determined in *p53* siRNA-transfected H9c2 cells coincubated with 5 mM fructose, 10 *μ*M pterostilbene, and 30 *μ*M allopurinol (*n* = 6). The relative miR-15b expression levels were normalized to U6. Cellular p-p53 protein levels were determined in miR-15b mimic (c) or miR-15b inhibitor (d)-transfected H9c2 cells coincubated with 5 mM fructose, 10 *μ*M pterostilbene, and 30 *μ*M allopurinol, respectively. Protein levels of TGF-*β*1, p-Smad2/3, and Smad4 were determined in these cells ((e–h) miR-15b mimic; (i–l) miR-15b inhibitor) (*n* = 6), respectively. Relative protein levels of p-p53 were normalized to p53, of TGF-*β*1 and Smad4 were normalized to GAPDH, and of p-Smad2/3 were normalized to Smad2/3, respectively. Data are expressed as the mean ± S.E.M.^#^*P* < 0.05, ^##^*P* < 0.01, and ^###^*P* < 0.001*vs.* normal cell control group; ^∗^*P* < 0.05, ^∗∗^*P* < 0.01, and ^∗∗∗^*P* < 0.01*vs.* fructose-vehicle cell group or fructose-vehicle+*p53* siRNA or miR-15b mimic or miR-15b inhibitor control cell group.

Furthermore, *p53* siRNA blocked fructose-induced cellular p-p53 upregulation, but not miR-15b expression change in H9c2 cells (Figures 5(a) and 5(b)) compared with the fructose-vehicle cell group. However, miR-15b mimic prevented fructose-induced p-p53 upregulation in H9c2 cells (Figure 5(c)), whereas p-p53 upregulation was observed in miR-15b inhibitor-transfected H9c2 cells (Figure 5(d)). miR-15b mimic also prevented fructose-induced upregulation of TGF-*β*1, p-Smad2/3, and Smad4 in H9c2 cells (Figures 5(e)–5(h)). Meanwhile, miR-15b inhibitor further increased TGF-*β*1, p-Smad2/3, and Smad4 in H9c2 cells exposed to fructose (Figures 5(i)–5(l)). These results indicate that miR-15b low expression may activate p-p53-mediated TGF-*β*1/Smads signaling in fructose-induced myocardial fibrosis.

Pterostilbene and allopurinol significantly upregulated miR-15b expression in fructose-fed rat hearts (Figure 4(c)) and fructose-exposed H9c2 cells (Figure 4(d)). In *p53* siRNA-transfected H9c2 cells, pterostilbene and allopurinol blocked fructose-induced change of cellular miR-15b low expression (Figure 5(b)) compared with fructose-vehicle and *p53* siRNA control cell group. Pterostilbene and allopurinol restored fructose-induced p-p53 upregulation in H9c2 cells under the transfection of miR-15b inhibitor (Figure 5(d)), but not miR-15b mimic (Figure 5(c)). Additionally, pterostilbene and allopurinol blocked fructose-induced upregulation of TGF-*β*1, p-Smad2/3, and Smad4 in H9c2 cells transfected with miR-15b inhibitor (Figures 5(i)–5(l)), but not miR-15b mimic (Figures 5(e)–5(h)), respectively. These results indicate that pterostilbene and allopurinol may reduce miR-15b-mediated p-p53 upregulation and then suppress TGF-*β*1/Smads signaling activation in fructose-incubated myocardial cells.

### 3.5. Pterostilbene Reduces Fructose-Induced Cardiac ROS to Block Pitx2c-Mediated miR-15b Low Expression in Cardiomyocytes with the Suppression of p-p53-Activated TGF-*β*1/Smads Signaling

**Figure 6:**
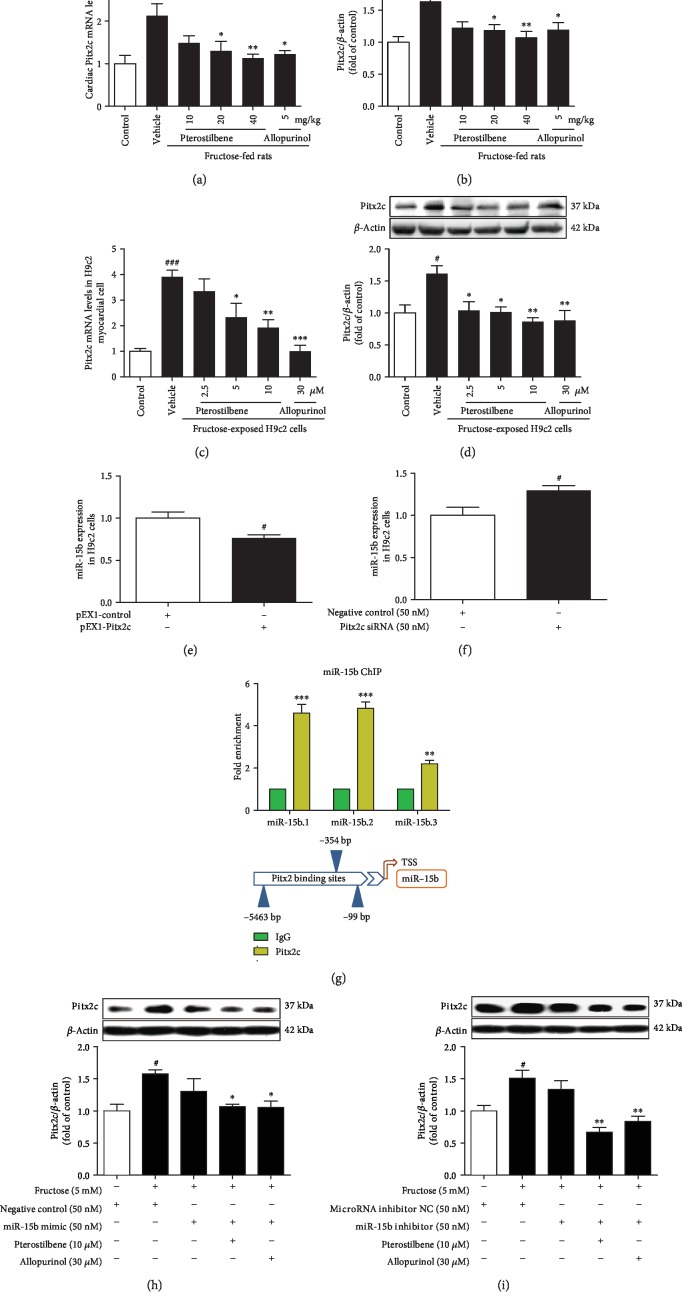
Pterostilbene and allopurinol downregulate Pitx2c to upregulate miR-15b expression in fructose-fed rat hearts and fructose-exposed H9c2 cells. Pitx2c mRNA and protein levels were measured in fructose-fed rat hearts (a, b) and fructose-exposed H9c2 cells (c, d) (*n* = 6), respectively. The relative Pitx2c mRNA levels were normalized to GAPDH. To detect whether Pitx2c mediated miR-15b expression, cellular miR-15b expression was determined in H9c2 cells transfected with pEX1-Pitx2c plasmid (e) or *Pitx2c* siRNA (f) (*n* = 6). Pitx2c bounds to DNA region upstream of the miR-15b genetic loci, and there was an observed enrichment in Pitx2c binding to miR-15b in H9c2 cells by the ChIP assay (g). Cellular Pitx2c protein levels were determined in miR-15b mimic (h) or miR-15b inhibitor (i)-transfected H9c2 cells coincubated with 5 mM fructose, 10 *μ*M pterostilbene, and 30 *μ*M allopurinol (*n* = 6). The relative miR-15b expression levels were normalized to U6. Relative protein levels of Pitx2c were normalized to *β*-actin. Data are expressed as the mean ± S.E.M.^#^*P* < 0.05, ^##^*P* < 0.01, and ^###^*P* < 0.001*vs.* normal animal control group, normal cell control group, or negative control cell group; ^∗^*P* < 0.05, ^∗∗^*P* < 0.01, and ^∗∗∗^*P* < 0.001*vs.* fructose-vehicle animal group, fructose-vehicle cell group, IgG-negative control group, or fructose-vehicle+miR-15b mimic or miR-15b inhibitor control cell group.

Pitx2c mRNA and protein levels were significantly increased in fructose-fed rat hearts (Figures 6(a) and 6(b)) and fructose-exposed H9c2 cells compared with the control group (Figures 6(c) and 6(d)). As shown in Figures 6(e) and 6(f), low miR-15b expression displayed significantly after Pitx2c overexpression, whereas *Pitx2c* siRNA caused miR-15b high expression in H9c2 cells compared with the negative control cell group, supporting negative regulation of miR-15b expression by Pitx2c.

Next, we screened potential conserved Pitx2c binding sites upstream of the miR-15b genetic loci. Target genes in the most enriched bins were further analyzed for the presence and the evolutionary conservation of Pitx2c consensus binding sequence, TAATCY (namely, TAATCC or TAATCT), on the -20 kb, intronic, and coding gene sequences [43]. Three conserved Pitx2c binding sites were identified -6 kb upstream of the miR-15b genetic loci. The ChIP assay showed that exogenous Pitx2c bounds to the all-putative binding sites upstream of the miR-15b genetic loci compared with the IgG-negative control group in H9c2 cells (Figure 6(g)). Additionally, miR-15b mimic or miR-15b inhibitor failed to alter fructose-induced Pitx2c overexpression in H9c2 cells compared with the fructose-vehicle cell group (Figures 6(h) and 6(i)), suggesting the involvement of Pitx2c/miR-15b pathway in myocardial cells under high fructose induction.

**Figure 7:**
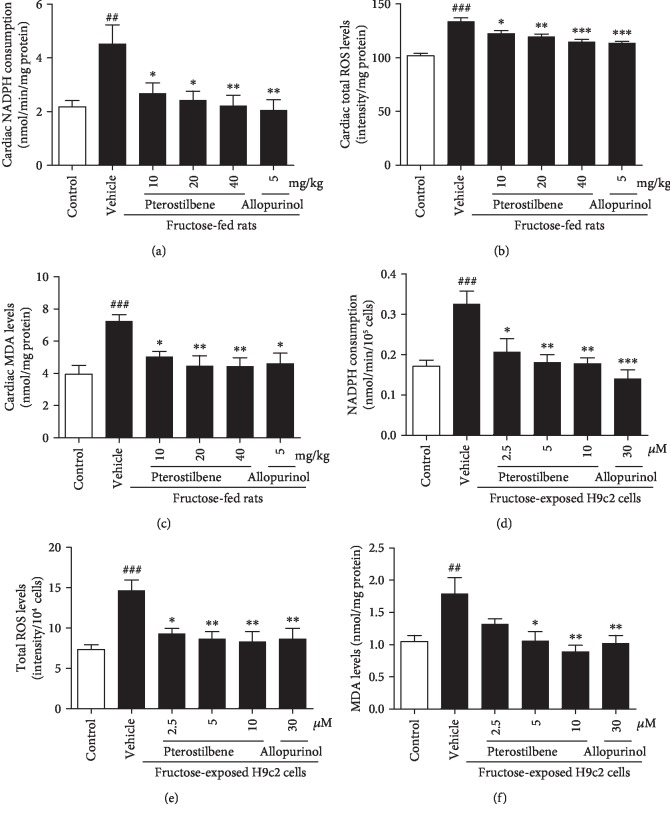
Effects of pterostilbene and allopurinol on oxidative stress in fructose-fed rat hearts and fructose-exposed H9c2 cells. NADPH oxidase activity, ROS production, and MDA levels were measured in fructose-fed rat hearts (a–c) and fructose-exposed H9c2 cells (d–f) (*n* = 6), respectively. Data are expressed as the mean ± S.E.M.^##^*P* < 0.01, ^###^*P* < 0.001*vs.* normal animal control group or normal cell control group; ^∗^*P* < 0.05, ^∗∗^*P* < 0.01, and ^∗∗∗^*P* < 0.001*vs.* fructose-vehicle animal group or fructose-vehicle cell group.

**Figure 8:**
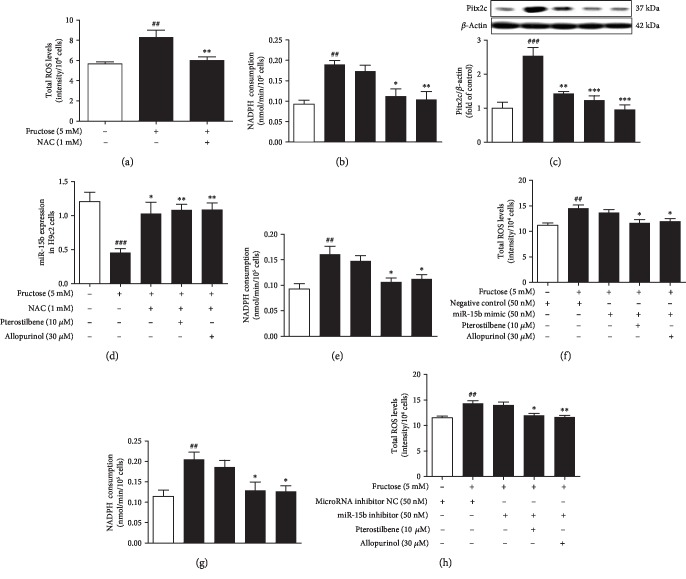
Pterostilbene and allopurinol reduce fructose-induced cardiac ROS to block Pitx2c-mediated miR-15b low expression in H9c2 cells. Cellular ROS production (a), NADPH oxidase activity (b), Pitx2c protein (c), and miR-15b expression (d) were determined in NAC-pretreated H9c2 cells coincubated with 5 mM fructose, 10 *μ*M pterostilbene, and 30 *μ*M allopurinol (*n* = 6), respectively. Cellular NADPH oxidase activity and ROS production were determined in miR-15b mimic (e, f) or miR-15b inhibitor (g, h)-transfected H9c2 cells (*n* = 6). Relative protein levels of Pitx2c were normalized to *β*-actin. The relative miR-15b expression levels were normalized to U6. Data are expressed as the mean ± S.E.M.^#^*P* < 0.05, ^##^*P* < 0.01, and ^###^*P* < 0.001*vs.* normal cell control group; ^∗^*P* < 0.05, ^∗∗^*P* < 0.01, and ^∗∗∗^*P* < 0.001*vs.* fructose-vehicle cell group or fructose-vehicle+NAC or miR-15b mimic or miR-15b inhibitor control cell group.

Oxidant stress was observed in fructose-fed rat hearts (Figures 7(a)–7(c)) and fructose-exposed H9c2 cells (Figures 7(d)–7(f)), showing hyperactivity of NADPH oxidase and overproduction of ROS and MDA. Compared with the fructose-vehicle cell group, NAC prevented fructose-induced ROS overproduction, but not NADPH oxidase hyperactivity in H9c2 cells (Figures 8(a) and 8(b)). Indeed, NAC prevented fructose-induced expression alteration of Pitx2c and miR-15b (Figures 8(c) and 8(d)), as well as p-p53, TGF-*β*1, p-Smad2/3, and Smad4 ([Supplementary-material supplementary-material-1]) in H9c2 cells. However, NADPH oxidase hyperactivity and ROS overproduction were still observed in miR-15b mimic or miR-15b inhibitor-transfected H9c2 cells exposed to fructose compared with the fructose-vehicle cell group (Figures 8(e)–8(h)). *p53* siRNA failed to alter fructose-induced NADPH oxidase hyperactivity, ROS overproduction, and Pitx2c overexpression ([Supplementary-material supplementary-material-1]) in H9c2 cells. Together, these results indicate that fructose may induce myocardial fibrosis, at least in part, by increasing cardiac ROS to drive Pitx2c-mediated miR-15b in myocardial cells.

Pterostilbene and allopurinol significantly suppressed NADPH oxidase hyperactivity, ROS overproduction, and MDA accumulation in the heart of fructose-fed rats (Figures 7(a)–7(c)) and fructose-exposed H9c2 cells (Figures 7(d)–7(f)), respectively. They remarkably downregulated Pitx2c mRNA and protein levels in these animal and cell models (Figures 6(a)–6(d)). In miR-15b mimic or miR-15b inhibitor-transfected H9c2 cells, pterostilbene and allopurinol reduced fructose-induced NADPH oxidase hyperactivity, ROS overproduction (Figures 8(e)–8(h)), and Pitx2c overexpression (Figures 6(h) and 6(i)) compared with fructose-vehicle and miR-15b mimic or miR-15b inhibitor control cell group. Moreover, they reduced fructose-induced NADPH oxidase hyperactivity, but not miR-15b downregulation, and Pitx2c overexpression in NAC-pretreated H9c2 cells (Figures 8(b)–8(d)). Additionally, pterostilbene and allopurinol failed to affect fructose-induced change of p-p53, TGF-*β*1, p-Smad2/3, and Smad4 in H9c2 cells pretreated with NAC ([Supplementary-material supplementary-material-1]). In *p53* siRNA-transfected H9c2 cells, they blocked fructose-induced NADPH oxidase hyperactivity, ROS overproduction, and Pitx2c overexpression ([Supplementary-material supplementary-material-1]) compared with fructose-vehicle and *p53* siRNA control cell group. These data indicate that pterostilbene and allopurinol may reduce ROS to block Pitx2c-mediated miR-15b low expression, subsequently inhibiting p-p53-activated TGF-*β*1/Smads signaling in fructose-induced myocardial fibrosis.

### 3.6. Pterostilbene Inhibits TGF-*β*1-Mediated CTGF Expression to Increase ANP, *α*-SMA, and FSP-1 in the Attenuation of Fructose-Induced Cardiomyocyte Hypertrophy and Fibrosis

*CTGF* siRNA was found to block fructose-induced change of ANP, *α*-SMA, and FSP-1 in H9c2 cells ([Supplementary-material supplementary-material-1]), while *TGF-β1* siRNA blocked fructose-induced change of CTGF as well as ANP, *α*-SMA, and FSP-1 in H9c2 cells ([Supplementary-material supplementary-material-1]). NAC, *Pitx2c* siRNA, or miR-15b mimic also prevented fructose-induced upregulation of CTGF, ANP, *α*-SMA, and FSP-1 in H9c2 cells ([Supplementary-material supplementary-material-1]), respectively. However, *p53* siRNA only prevented fructose-induced increase of CTGF, ANP, and *α*-SMA but had no significant change of FSP-1 expression ([Supplementary-material supplementary-material-1]). These observations indicate that TGF-*β*1 signaling activation may increase CTGF expression, causing hypertrophic and fibrotic response under fructose induction.

In *CTGF* siRNA-transfected H9c2 cells, pterostilbene and allopurinol failed to alter fructose-induced change of cellular ANP, *α*-SMA, and FSP-1 ([Supplementary-material supplementary-material-1]). In NAC, *Pitx2c* siRNA, miR-15b mimic, or *TGF-β1* siRNA-transfected H9c2 cells, pterostilbene, and allopurinol had no effect on expression change of cellular CTGF, ANP, *α*-SMA, and FSP-1 under fructose exposure (Figures [Supplementary-material supplementary-material-1] and [Supplementary-material supplementary-material-1]). In *p53* siRNA-transfected H9c2 cells, pterostilbene and allopurinol inhibited fructose-induced change of cellular FSP-1, but not CTGF, ANP, and *α*-SMA ([Supplementary-material supplementary-material-1]). These data indicate that pterostilbene and allopurinol may reduce TGF-*β*1-mediated CTGF to suppress ANP, *α*-SMA, and FSP-1 overexpression in fructose-induced myocardial hypertrophy and fibrosis.

## 4. Discussion

Emerging evidence suggests that myocardial fibrosis may impair myocyte contractility, disrupt electrical coupling, and eventually develop left ventricular remodeling, diastolic dysfunction, and heart failure [1–3, 44]. No specific therapeutic strategy is available to recover heart function in myocardial fibrosis. Excessive fructose intake induces cardiomyocyte hypertrophy and myocardial fibrosis [1, 2, 17], but the underlying molecular mechanisms remain unclear. Using fructose-exposed rat and H9c2 cell models, we provided the evidence that ROS induced Pitx2c-mediated miR-15b low expression and provoked p-p53-dependent TGF-*β*1/Smads signaling activation, subsequently promoting CTGF-mediated myocardial fibrosis. More importantly, pterostilbene and allopurinol with antioxidant capacity downregulated Pitx2c to increase miR-15b expression and suppressed p-p53 to reduce TGF-*β*1/Smads signaling activation and CTGF expression, resulting in the attenuation of fructose-induced myocardial fibrosis.

With respect to myocardial injury, cardiac biomarkers CK, CK-MB, MB, and cTn-T levels in serum were significantly increased in fructose-fed rats, being consistent with the previous reports [45, 46]. Fructose induced-cardiomyocyte hypertrophy and fibrosis were also observed in rats. Importantly, this study showed that p-p53 upregulation drove TGF-*β*1/Smads signaling activation in the hearts and cardiomyocytes under fructose stimulation. It is noteworthy that overexpression of p53 and TGF-*β*1 is detected in the heart of high oxygen-exposed rats with cardiomyocyte hypertrophy and fibrosis [15]. These results indicate that p-p53 upregulation in myocardial cells may induce myocardial fibrosis by activating TGF-*β*1/Smads signaling.

Osteoblastic specific miR-15b is predicted to target 16 genes in p53 signaling pathway identified by the bioinformatics approach [13]. In this study, fructose was found to induce a significant downregulation of miR-15b expression in plasma and heart of rats as well as in myocardial cells, which were consistent with the recent reports in plasma and heart of diabetic patients and mice [12]. In case of fructose-induced myocardial fibrosis, there was evidence of miR-15b low expression-mediated p-p53 to activate TGF-*β*1/Smads signaling. High fructose triggered miR-15b low expression, which may be a risk factor in myocardial fibrosis. Continuous monitoring of miR-15b levels in plasma may be considered as a noninvasive biomarker for cardiac fibrotic remodeling identification in patients.

Recent study has revealed the negative regulation of miR-15b by Pitx2c on the transcription level [21]. Pitx2c is significantly reactivated in the left ventricular myocardium of patients with systolic heart failure [47] and myocardial injury [20]. High Pitx2c expression is detected in atrial myocytes or atrial appendages in chronic atrial fibrillation patients [48, 49]. Here, we showed significant Pitx2 overexpression in fructose-induced myocardial fibrosis of rats and cells. We hypothesized that targeting Pitx2c and miR-15b might alleviate myocardial fibrosis under fructose induction. We further supported Pitx2c-mediated negative regulation of miR-15b expression in fructose-stimulated myocardial cells. More importantly, exogenous Pitx2c bounds to the all-putative binding sites upstream of the miR-15b genetic loci. These results provide an evidence that Pitx2c/miR-15b pathway possibly mediates fructose-induced myocardial fibrosis.

Pitx2 has been demonstrated to promote heart repair by activating antioxidant response [20]. High-fat and high-fructose diet increases myocardial ROS production and oxidative stress, cardiomyocyte hypertrophy, interstitial fibrosis, and left ventricular diastolic dysfunction in mice [34]. Our previous study showed that fructose-induced ROS was a major initiator of myocardial damage in rats with myocardial fibrosis [17]. In this study, ROS inhibitor prevented fructose-induced Pitx2c upregulation and miR-15b low expression, as well as p-p53-activated TGF-*β*1/Smads signaling in myocardial cells. These observations indicate that fructose-triggered cardiac ROS may be the primary step driving Pitx2c upregulation to reduce miR-15b expression, providing a novel mechanistic insight into the link between oxidative stress and myocardial fibrosis. Thus, ROS-driven Pitx2c/miR-15b pathway was required for p-p53-dependent TGF-*β*1/Smads signaling activation in myocardial fibrosis. Pitx2c may be a novel prominent target in the development of fructose-induced myocardial fibrosis for further studies.

Fructose feeding directly increases TGF-*β*1, *α*-SMA, and FSP-1 expression via ROS overload in mouse hearts and primary cardiac myocytes [10]. High CTGF protein levels are detected in fructose-fed rats with cardiac fibrosis [50]. ROS-dependent CTGF overproduction is observed in haemodynamic stress-induced myocardial fibrosis in mice [51]. Expression of TGF-*β*1 and CTGF is significantly upregulated in hearts of myocardial infarction rats and cardiac ischemia patients [4]. Of note, TGF-*β*1 induces CTGF expression by activating its promoter in rat primary cardiac myocytes [4]. Human recombinant CTGF induces *α*-SMA and FSP-1 expression in skin-derived precursors into fibroblast-like cells [52]. Its specific deletion in smooth muscle cell fibroblast reduces *α*-SMA and FSP-1 in Ang II-induced skin fibrosis of C57BL/6J mice [53]. *TGF-β1* or *CTGF* siRNA also inhibits hypertrophic marker ANP expression in phenylephrine-induced primary rat cardiomyocyte hypertrophy [54]. The current study showed that TGF-*β*1-mediated upregulation of prohypertrophic and profibrotic markers ANP, *α*-SMA, FSP-1, and CTGF might be due to ROS-driven Pitx2c/miR-15b pathway in fructose-exposed H9c2 cells. Of note, *p53* siRNA inhibited ANP, *α*-SMA, and CTGF upregulation but had no significant effect on FSP-1 *in vitro*. These results indicate that FSP-1 upregulation may not entirely depend on this signaling under fructose stimulation. Critically, CTGF blockade reduced ANP, *α*-SMA, and FSP-1 expression in fructose-exposed H9c2 cells, suggesting that CTGF may modulate TGF-*β*1 signaling in response to fructose. The abnormal expression of CTGF could be employed as a diagnostic marker for fructose-induced myocardial hypertrophy and fibrosis.

Pterostilbene is reported to reduce NADPH oxidase-dependent superoxide anion production and oxidative stress and prevent right ventricle hypertrophy in monocrotaline-induced pulmonary hypertension of rats [24]. It also decreases oxidative stress and activates p53-evoked apoptosis in mice [27]. Moreover, pterostilbene inhibits TGF-*β*1/Smads signaling and alleviates dimethylnitrosamine-induced liver fibrosis in rats [28] and prevents against myocardial ischemia/reperfusion injury in rats [26]. In this study, we firstly reported that pterostilbene and allopurinol with antioxidant capacity potentially downregulated Pitx2c to increase miR-15b expression and hence reduced p-p53-dependent TGF-*β*1/Smads signaling activation, being consistent with the alleviation of fructose-induced myocardial fibrosis. Our results thereby suggest that Pitx2c-mediated miR-15b may be the therapeutic target in the alleviation of high fructose diet-induced myocardial fibrosis. Additionally, pterostilbene and allopurinol diminished TGF-*β*1-mediated CTGF upregulation, inhibiting the hypertrophic and fibrotic response in these animal and cell models. Thus, specific agonist of CTGF may offer a new therapeutic strategy to prevent myocardial hypertrophy and fibrosis.

The safety of pterostilbene has been confirmed in humans (up to 250 mg/day), without statistically significant major adverse drug reactions [55]. Based on our findings, the inclusion of blueberries and grape vines in the diet, especially offering a broader range of available pharmacological compound pterostilbene in subjects, may reduce the risk factors associated with myocardial diseases, comprising a more efficacious therapeutic option. On the other hand, the novel findings of this study may be of importance contributing to understanding the potential beneficial effects of allopurinol treatment in myocardial fibrosis.

## 5. Conclusion

In conclusion, the results from this study demonstrate that Pitx2c may be a novel factor participating in fructose-induced myocardial fibrosis. High fructose-triggered cardiac ROS may be the primary step accounting for Pitx2c upregulation to reduce miR-15b expression and then activating TGF-*β*1/Smads signaling in CTGF-mediated myocardial fibrosis ([Supplementary-material supplementary-material-1]). Pterostilbene and allopurinol with antioxidant activity downregulate Pitx2c and upregulate miR-15b and then suppress p-p53-dependent TGF-*β*1/Smads signaling activation to reduce CTGF, resulting in the alleviation of fructose-induced myocardial fibrosis. Thus, this study suggests that inhibition of Pitx2c-mediated miR-15b pathway by pterostilbene and allopurinol may provide a novel therapeutic strategy for myocardial fibrosis associated with excess fructose consumption.

## Data Availability

The data used to support the findings of this study are included within the article.

## Abbreviations

ANOVA: Analysis of variance
ANP: Atrial natriuretic peptide
*α*-SMA: Alpha smooth muscle-actin
*β*-MHC: Beta myosin heavy chain
BNP: Brain natriuretic peptide
ChIP: Chromatin immunoprecipitation
CK: Creatine kinase
CK-MB: Creatine kinase isoenzyme
CTGF: Connective tissue growth factor
cTn-T: Troponin
FISH: Fluorescence in situ hybridization
FSP-1: Fibroblast specific-1
HW/BW: Heart-to-body weight
IF: Immunofluorescence
MB: Myoglobin
MDA: Malondialdehyde
miR-15b: MicroRNA-15b
NAC: N-Acetylcysteine
NADPH: Nicotinamide adenine dinucleotide phosphate
Pitx2: Paired-like homeodomain 2
p-p53: p53 phosphorylation
ROS: Reactive oxygen species
Smads: (Small) mothers against decapentaplegic homologs
TGF-*β*1: Transforming growth factor-*β*1.

## Supplementary Materials

Supplementary Materials Table S1: aberrantly expressed miRNAs in the fructose-vehicle animal group revealed by a microarray scan. Table S2: primer, siRNA, and miRNA sequences. Table S3: Pitx2c gene sequences inserted in the plasmid expression vector pEX1-Pitx2c were listed as follows. Table S4: the miR-15b promoter sequences containing the Pitx2c binding sites were amplified by qRT-PCR using the following primers. Figure S1: the transfection efficiency of p53 gene silencing in H9c2 cells. p53 mRNA levels were assayed in 50 nM p53 siRNA- and negative control-transfected H9c2 cells (*n* = 6), respectively. Data are expressed as the mean ± S.E.M.^#^*P* < 0.05 vs. negative control cell group. Figure S2: the transfection efficiency of CTGF gene silencing in H9c2 cells. CTGF mRNA levels were assayed in 50 nM CTGF siRNA- and negative control-transfected H9c2 cells (*n* = 6), respectively. Data are expressed as the mean ± S.E.M.^#^*P* < 0.05 vs. negative control cell group. Figure S3: the transfection efficiency of TGF-*β*1 gene silencing in H9c2 cells. TGF-*β*1 mRNA levels were assayed in 50 nM TGF-*β*1 siRNA- and negative control-transfected H9c2 cells (*n* = 6), respectively. Data are expressed as the mean ± S.E.M.^#^*P* < 0.05 vs. negative control cell group. Figure S4: the transfection efficiency of miR-15b gene overexpression in H9c2 cells. miR-15b expression levels were assayed in 50 nM miR-15b mimic- and negative control-transfected H9c2 cells (*n* = 6), respectively. Data are expressed as the mean ± S.E.M.^###^*P* < 0.001 vs. negative control cell group. Figure S5: the transfection efficiency of miR-15b gene silencing in H9c2 cells. miR-15b expression levels were assayed in 50 nM miR-15b inhibitor- and microRNA inhibitor NC-transfected H9c2 cells (*n* = 6), respectively. Data are expressed as the mean ± S.E.M.^#^*P* < 0.05 vs. microRNA 7 inhibitor NC cell group. Figure S6: the transfection efficiency of Pitx2c gene overexpression in H9c2 cells. Pitx2c mRNA levels were assayed in pEX1-Pitx2c plasmid- and pEX1-control plasmid-transfected H9c2 cells (*n* = 6), respectively. Data are expressed as the mean ± S.E.M.^###^*P* < 0.001 vs. pEX1-control cell group. Figure S7: the transfection efficiency of Pitx2c gene silencing in H9c2 cells. Pitx2c mRNA levels were assayed in Pitx2c siRNA- and negative control-transfected H9c2 cells (*n* = 6), respectively. Data are expressed as the mean ± S.E.M.^#^*P* < 0.05 vs. negative control cell group. Figure S8: RNA polymerase II occupancy in the GAPDH promoter in H9c2 cells by ChIP-qRT-PCR assays. There was an observed enrichment in RNA polymerase II binding GAPDH promoter (*n* = 3) after pEX1-Pitx2c plasmid-transfected H9c2 cells. Data are expressed as the mean ± S.E.M.^∗∗∗^*P* < 0.001 vs. IgG-negative control group. Figure S9: effects of pterostilbene and allopurinol on fructose-induced alteration of cellular p-p53 and TGF-*β*1/Smads signaling in NAC-pretreated H9c2 cells. Cellular p-p53 (A), TGF-*β*1 (B), p-Smad2/3 (C-D), and Smad4 (E) protein levels were determined in NAC-pretreated H9c2 cells coincubated with 5 mM fructose, 10 *μ*M pterostilbene, and 30 *μ*M allopurinol (*n* = 6), respectively. Relative protein levels of p-p53 were normalized to p53, respectively. The relative protein levels of TGF-*β*1 and Smad4 were normalized to GAPDH, respectively (*n* = 6). Relative protein levels of 8 p-Smad2/3 were normalized to Smad2/3, respectively (*n* = 6). Data are expressed as the mean ± S.E.M.^#^*P* < 0.05, ^##^*P* < 0.01, and ^###^*P* < 0.001 vs. normal cell control group; ^∗^*P* < 0.05, ^∗∗^*P* < 0.01, and ^∗∗∗^*P* < 0.001 vs. fructose-vehicle cell group or fructose-vehicle+NAC control cell group. Figure S10: effects of pterostilbene and allopurinol on fructose-induced alteration of cellular NADPH oxidase activity, ROS production, and Pitx2c protein in p53 siRNA-transfected H9c2 cells. Cellular NADPH oxidase activity (A), ROS production (B), and Pitx2c protein levels (C) were determined in p53 siRNA-transfected H9c2 cells coincubated with 5 mM fructose, 10 *μ*M pterostilbene, and 30 *μ*M allopurinol (*n* = 6). Relative protein levels of Pitx2c were normalized to *β*-actin, respectively. Data are expressed as the mean ± S.E.M.^##^*P* < 0.01 vs. normal cell control group; ^∗^*P* < 0.05 vs. fructose-vehicle cell group or fructose-vehicle+p53 siRNA control cell group. Figure S11: pterostilbene and allopurinol decrease TGF-*β*1-mediated CTGF expressions to inhibit downstream hypertrophic and fibrotic response-associated indicator ANP, *α*-SMA, and FSP-1 expressions in fructose-exposed H9c2 cells. Cellular protein levels of ANP (A), *α*-SMA (B), and FSP-1 (C) were determined in CTGF siRNA-transfected H9c2 cells coincubated with 5 mM fructose, 10 *μ*M pterostilbene, and 30 *μ*M allopurinol (*n* = 6). Cellular protein levels of CTGF (D), ANP (E), *α*-SMA (F), and FSP-1 (G) were determined in TGF-*β*1 siRNA-transfected H9c2 cells coincubated with 5 mM fructose, 10 *μ*M pterostilbene, and 30 *μ*M allopurinol (*n* = 6). Relative protein levels of CTGF, ANP, and FSP-1 were normalized to *β*-actin and of *α*-SMA were normalized to GAPDH, respectively. Data are expressed as the mean ± S.E.M.^#^*P* < 0.05, ^##^*P* < 0.01 vs. normal cell control group; ^∗^*P* < 0.05, ^∗∗^*P* < 0.01 vs. fructose-vehicle cell group or fructose-vehicle+CTGF siRNA or 9 TGF-*β*1 siRNA control cell group. Figure S12: effects of pterostilbene and allopurinol on fructose-induced alteration of cellular CTGF, ANP, *α*-SMA, and FSP-1 in NAC-pretreated, Pitx2c siRNA, miR-15b mimic, and p53 siRNA-transfected H9c2 cells. Cellular CTGF, ANP, *α*-SMA, and FSP-1 protein levels were determined in NAC (A-D)-pretreated, Pitx2c siRNA (E-H), miR-15b mimic (I-L), and p53 siRNA (M-P)-transfected H9c2 cells coincubated with 5 mM fructose, 10 *μ*M pterostilbene, and 30 *μ*M allopurinol, respectively. Relative protein levels of CTGF, ANP, and FSP-1 were normalized to *β*-actin and of *α*-SMA were normalized to GAPDH, respectively. Data are expressed as the mean ± S.E.M.^#^*P* < 0.05, ^##^*P* < 0.01, and ^###^*P* < 0.001 vs. normal cell control group; ^∗^*P* < 0.05, ^∗∗^*P* < 0.01, and ^∗∗∗^*P* < 0.01 vs. fructose-vehicle cell group or fructose-vehicle+NAC or Pitx2c siRNA or miR-15b mimic or p53 siRNA control cell group. Figure S13: proposed scheme of the mechanisms underlying fructose-induced myocardial fibrosis, as well as the attenuation of pterostilbene and allopurinol. High fructose triggers cardiac ROS to increase Pitx2c and then reduce miR-15b expression; this event upregulates p-p53 to activate TGF-*β*1/Smads signaling in myocardial fibrosis. Pterostilbene and allopurinol with the reduction of ROS downregulate Pitx2c to increase miR-15b expression and then suppress p-p53-mediated TGF-*β*1/Smads signaling activation, resulting in the alleviation of fructose-induced myocardial fibrosis.
