# Supplementary material for: Pterostilbene Attenuates Fructose-Induced Myocardial Fibrosis by Inhibiting ROS-Driven Pitx2c/miR-15b Pathway
Source: Oxid Med Cell Longev. 2019 Dec 4;2019:1243215. doi: 10.1155/2019/1243215 (PMC6913258; doi:10.1155/2019/1243215)
Supplement: Supplementary Materials — Table S1: aberrantly expressed miRNAs in the fructose-vehicle animal group revealed by a microarray scan. Table S2: primer, siRNA, and miRNA sequences. Table S3: Pitx2c gene sequences inserted in the plasmid expression vector pEX1-Pitx2c were listed as follows. Table S4: the miR-15b promoter sequences containing the Pitx2c binding sites were amplified by qRT-PCR using the following primers. Figure S1: the transfection efficiency of p53 gene silencing in H9c2 cells. p53 mRNA levels were assayed in 50 nM p53 siRNA- and negative control-transfected H9c2 cells (n = 6), respectively. Data are expressed as the mean ± S.E.M.#P < 0.05 vs. negative control cell group. Figure S2: the transfection efficiency of CTGF gene silencing in H9c2 cells. CTGF mRNA levels were assayed in 50 nM CTGF siRNA- and negative control-transfected H9c2 cells (n = 6), respectively. Data are expressed as the mean ± S.E.M.#P < 0.05 vs. negative control cell group. Figure S3: the transfection efficiency of TGF-β1 gene silencing in H9c2 cells. TGF-β1 mRNA levels were assayed in 50 nM TGF-β1 siRNA- and negative control-transfected H9c2 cells (n = 6), respectively. Data are expressed as the mean ± S.E.M.#P < 0.05 vs. negative control cell group. Figure S4: the transfection efficiency of miR-15b gene overexpression in H9c2 cells. miR-15b expression levels were assayed in 50 nM miR-15b mimic- and negative control-transfected H9c2 cells (n = 6), respectively. Data are expressed as the mean ± S.E.M.###P < 0.001 vs. negative control cell group. Figure S5: the transfection efficiency of miR-15b gene silencing in H9c2 cells. miR-15b expression levels were assayed in 50 nM miR-15b inhibitor- and microRNA inhibitor NC-transfected H9c2 cells (n = 6), respectively. Data are expressed as the mean ± S.E.M.#P < 0.05 vs. microRNA 7 inhibitor NC cell group. Figure S6: the transfection efficiency of Pitx2c gene overexpression in H9c2 cells. Pitx2c mRNA levels were assayed in pEX1-Pitx2c plasmid- and pEX1-control plasmid [file 1243215.f1.pdf]

Pterostilbene attenuates fructose-induced myocardial fibrosis by inhibiting ROS-driven Pitx2c/miR-15b pathway

Pterostilbene protects from myocardial fibrosis

Lin-Lin Kang[1], Dong-Mei Zhang[1], Rui-Qing Jiao[1], Shu-Man Pan[1], Xiao-Juan Zhao[1], Yan-Jing Zheng[1], Tian-Yu Chen[1,2], and Ling-Dong Kong[1]

[1]State Key Laboratory of Pharmaceutical Biotechnology, School of Life Science, Nanjing University, Nanjing, P. R. China

[2]State Key Laboratory Cultivation Base for TCM Quality and Efficacy, Nanjing University of Chinese Medicine, Nanjing 210023, P. R. China

Correspondence and requests for materials should be addressed to Dr. Ling-Dong Kong (Tel. and fax: +86-25-83594691; email: kongld@nju.edu.cn)

The authors declare no conflict of interest.

Supplementary Table

Table S1. Aberrantly expressed miRNAs in fructose-vehicle animal group revealed by a microarray scan.

**Name**	**Fold change**	***p*-value**
**Positive Genes:**	**Fold change ≥ 1.5**	
rno-miR-99b-3p	2.063641	0.481478
rno-miR-493-5p	1.97534	0.101411
rno-let-7a-5p	1.849243	0.177056
rno-miR-465-5p	1.772754	0.121411
rno-miR-7b	1.742648	0.154412
rno-miR-9b-5p	1.728478	0.16723
rno-miR-743a-3p	1.720878	0.192757
rno-miR-187-5p	1.701578	0.338445
rno-miR-181b-5p	1.681387	0.196039
rno-miR-433-5p	1.674082	0.068219
rno-miR-29b-3p	1.642932	0.184706
rno-miR-381-3p	1.62233	0.271121
rno-miR-365-3p	1.605889	0.19909
rno-miR-347	1.572813	0.130232
rno-miR-598-3p	1.531271	0.330219
rno-miR-352	1.523969	0.193969
rno-miR-136-5p	1.522933	0.189312
rno-miR-324-3p	1.506851	0.257809
rno-miR-30a-5p	1.504091	0.202055
**Name**	**Fold change**	***p*-value**
**Negative Genes:**	**Fold change ≤ 0.6**	
rno-miR-674-3p	0.591335	0.203443
rno-let-7e-3p	0.577555	0.083248
rno-miR-380-5p	0.565473	0.167421
rno-miR-15b-5p	0.564152	0.050515
rno-miR-30b-3p	0.563457	0.308952
rno-miR-337-5p	0.557398	0.258185
rno-miR-448-5p	0.553514	0.260916
rno-miR-320-3p	0.542009	0.33094
rno-miR-142-5p	0.540299	0.11335
rno-miR-1843a-5p	0.538865	0.364358
rno-miR-98-3p	0.5333	0.069132
rno-miR-181c-3p	0.52443	0.209769
rno-miR-3591	0.523418	0.295768
rno-miR-20a-5p	0.497283	0.065369
rno-miR-758-3p	0.4873	0.286007
rno-miR-339-5p	0.487154	0.116773
rno-miR-150-5p	0.482113	0.368901
rno-miR-370-3p	0.474309	0.21584
rno-miR-935	0.449092	0.377685
rno-miR-212-5p	0.448323	0.297019
rno-miR-1224	0.432602	0.320107
rno-miR-331-5p	0.416514	0.339699
rno-miR-223-3p	0.40993	0.032337
rno-let-7f-1-3p	0.398882	0.217077
rno-miR-155-3p	0.301484	0.288519

Herein, t-test analysis generated a list of miRNAs that were differentially expressed in the FM (normal control animal group) samples compared to the FC (fructose-vehicle animal group) samples (n=3).

Table S2. primer, siRNA and miRNA sequences.

ID	Sense primer (5′→ 3′)	Antisense primer (5′→ 3′)
Pitx2c	GACTCCTCCAAACATAGACT	GATTTCTTCGCGTGTGGAC
p53	GGGAATGGGTTGGTAGTT	AGAGTGGAGGAAATGGGT
GAPDH	ACAACTTTGGTATCGTGGAAGG	GCCATCACGCCACAGTTTC
TGF-β1	CTAATGGTGGACCGCAACAAC	CACTGCTTCCCGAATGTCTGA
CTGF	ACCGCACAGAACCACCACACT	CCCTTACTCCCTGGCTTTACGC
miR-15b-5p	GCTAGCAGCACATCATGG	CAGCCACAAAAGAGCACAAT
miR-15b-Stem-loop	CCTGTTGTCTCCAGCCACAAAAGAGCACAATATTTCAGGAGACAACAGGTGTAAA	
U6	CTCGCTTCGGCAGCACA	AACGCTTCACGAATTTGCGT
URP	TGGTGTCGTGGAGTCG	
Pitx2c siRNA	CCGGGUUUGGUUCAAGAAUTT	AUUCUUGAACCAAACCCGGTT
	UCAACUCUAUGAACGUCAATT	UUGACGUUCAUAGAGUUGATT
	GCCUGAAUAACUUGAACAATT	UUGUUCAAGUUAUUCAGGCTT
p53 siRNA	CCUGUGCAGUUGUGGGUCATT	UGACCCACAACUGCACAGGTT
TGF-β1 siRNA	CUGAGUGGCUGUCUUUUGATT	UCAAAAGACAGCCACUCAGTT
CTGF siRNA	ACCUAGAGGAAAACAUUAATT	UUAAUGUUUUCCUCUAGGUTT
miR-15b mimic	UAGCAGCACAUCAUGGUUUACA	UAAACCAUGAUGUGCUGCUAUU
Negative control	UUCUCCGAACGUGUCACGUTT	ACGUGACACGUUCGGAGAATT
miR-15b inhibitor	UGUAAACCAUGAUGUGCUGCUA	
MicroRNA inhibitor NC	CAGUACUUUUGUGUAGUACAA	

Table S3. Pitx2c gene sequences inserted in the plasmid expression vector pEX1-Pitx2c were listed as follows.

ID	Pitx2c
sequences	ATGAACTGCATGAAAGGCCCGCTGCCCTTGGAGCACCGAGCAGCCG<br>GGACTAAGCTGTCGGCCGCTTCCTCACCCTTCTGTCACCATACCCAG<br>GCGTTAGCCATGGCTTCGGTCCTAGCTCCTGGTCAGCCCCGCTCCCTG<br>GACGCCTCCAAACATAGACTGGAGGTGCATACAATCTCCGATACGTC<br>CAGCCCTGAAGTCGCAGAGAAAGATAAGGGCCAGCAGGGAAAGAAT<br>GAGGATGTGGGCGCAGAGGACCCGTCCAAGAAGAAGCGGCAACGC<br>CGGCAGAGGACTCACTTTACTAGTCAGCAGCTCCAGGAGCTGGAAG<br>CCACTTTCCAGAGAAACCGCTACCCAGACATGTCCACTCGCGAAGAA<br>ATCGCCGTGTGGACCAACCTTACGGAAGCCCGAGTCCGGGTTTGGTT<br>CAAGAATCGCCGGGCCAAATGGAGAAAGCGGGAGCGCAACCAGCA<br>GGCCGAGCTGTGTAAGAATGGCTTCGGGCCGCAGTTCAATGGGCTCA<br>TGCAGCCGTACGATGACATGTACCCCGGCTACTCGTACAACAATTGG<br>GCTGCCAAGGGCCTCACGTCAGCGTCTCTGTCCACCAAGAGCTTCCC<br>CTTCTTCAACTCTATGAACGTCAATCCCCTATCCTCCCAGAGCATGTTT<br>TCCCCGCCCAACTCCATCTCATCCATGAGTATGTCGTCCAGCATGGTG<br>CCGTCCGCGGTGACCGGCGTCCCGGGCTCCAGCCTCAATAGCCTGAA<br>TAACTTGAACAACCTGAGCAGCCCGTCGCTGAATTCCGCGGTGCCCA<br>CGCCCGCCTGTCCTTACGCGCCGCCGACTCCTCCGTACGTTTATAGGG<br>ACACATGTAACTCGAGCCTGGCCAGTCTGAGACTAAAAGCAAAGCA<br>ACACTCCAGCTTCGGCTACGCCAGCGTGCAGAACCCGGCCTCCAACC<br>TGAGCGCTTGCCAGTATGCGGTCGACCGGCCGGTGTGA

Table S4. The miR-15b promoter sequences containing the Pitx2c binding sites were amplified by qRT-PCR using the following primers.

ID	Sense primer (5′→ 3′)	Antisense primer (5′→ 3′)
miR-15b.1	TTACTCACTAATCCCGGTTTC	TTGCGGAGCATCAGTTAT
miR-15b.2	ATGGTTGTCAGCTTATGGTC	CTGAAACTAATGGAACGGATT
miR-15b.3	CATGGGATTGACTTAGACCA	ATTCACCAAACAGAACAACG
GAPDH	ACGTAGCTCAGGCCTCAAGA	GCGGGCTCAATTTATAGAAAC

Supplementary Figures Legends

Figure S1. The transfection efficiency of p53 gene silencing in H9c2 cells. P53 mRNA levels were assayed in 50 nM *p53* siRNA- and negative control-transfected H9c2 cells (n=6), respectively. Data are expressed as the mean ± S.E.M. $^{\#}P < 0.05$ *vs* negative control cell group.

Figure S2.The transfection efficiency of CTGF gene silencing in H9c2 cells. CTGF mRNA levels were assayed in 50 nM *CTGF* siRNA- and negative control-transfected H9c2 cells (n=6), respectively. Data are expressed as the mean ±S.E.M. $^{\#}P < 0.05$ *vs* negative control cell group.

Figure S3. The transfection efficiency of TGF-β1 gene silencing in H9c2 cells. TGF-β1 mRNA levels were assayed in 50 nM *TGF-β1* siRNA- and negative control-transfected H9c2 cells (n=6), respectively. Data are expressed as the mean ±S.E.M. $^{\#}P < 0.05$ *vs* negative control cell group.

Figure S4. The transfection efficiency of miR-15b gene overexpression in H9c2 cells. MiR-15b expression levels were assayed in 50 nM miR-15b mimic- and negative control-transfected H9c2 cells (n=6), respectively. Data are expressed as the mean ± S.E.M. $^{\#\#\#}P < 0.001$ *vs* negative control cell group.

Figure S5. The transfection efficiency of miR-15b gene silencing in H9c2 cells. MiR-15b expression levels were assayed in 50 nM miR-15b inhibitor- and microRNA inhibitor NC-transfected H9c2 cells (n=6), respectively. Data are expressed as the mean ± S.E.M. $^{\#}P < 0.05$ *vs* microRNA

inhibitor N.C cell group.

Figure S6. **The transfection efficiency of Pitx2c gene overexpression in H9c2 cells.** Pitx2c mRNA levels were assayed in pEX1-Pitx2c plasmid- and pEX1-Control plasmid -transfected H9c2 cells (n=6), respectively. Data are expressed as the mean ± S.E.M. $^{###}P < 0.001$ *vs* pEX1-Control cell group.

Figure S7. **The transfection efficiency of Pitx2c gene silencing in H9c2 cells.** Pitx2c mRNA levels were assayed in *Pitx2c* siRNA- and negative control-transfected H9c2 cells (n=6), respectively. Data are expressed as the mean ± S.E.M. $^{#}P < 0.05$ *vs* negative control cell group.

Figure S8. **RNA polymerase II occupancy in the GAPDH promoter in H9c2 cells by ChIP-qRT-PCR assays.** There was an observed enrichment in RNA polymerase II binding GAPDH promoter (n=3) after pEX1-Pitx2c plasmid-transfected H9c2 cells. Data are expressed as the mean ± S.E.M. $^{***}P < 0.001$ *vs* IgG-negative control group.

Figure S9. **Effects of pterostilbene and allopurinol on fructose-induced alteration of cellular p-p53 and TGF-β1/Smads signaling in NAC pretreated-H9c2 cells.** Cellular p-p53 (A), TGF-β1 (B), p-Smad2/3 (C-D) and Smad4 (E) protein levels were determined in NAC-pretreated H9c2 cells co-incubated with 5 mM fructose, 10 μM pterostilbene and 30 μM allopurinol (n=6), respectively. Relative protein levels of p-p53 were normalized to p53, respectively. The relative protein levels of TGF-β1 and Smad4 were normalized to GAPDH, respectively (n=6). Relative protein levels of

p-Smad2/3 were normalized to Smad2/3, respectively (n=6). Data are expressed as the mean ± S.E.M. $^{\#}P < 0.05$, $^{\#\#}P < 0.01$, $^{\#\#\#}P < 0.001$ *vs* normal cell control group; $^{*}P < 0.05$, $^{**}P < 0.01$, $^{***}P < 0.001$ *vs* fructose-vehicle cell group, or fructose-vehicle + NAC control cell group.

Figure S10. **Effects of pterostilbene and allopurinol on fructose-induced alteration of cellular NADPH oxidase activity, ROS production and Pitx2c protein in *p53* siRNA transfected-H9c2 cells.** Cellular NADPH oxidase activity (A), ROS production (B) and Pitx2c protein levels (C) were determined in *p53* siRNA-transfected H9c2 cells co-incubated with 5 mM fructose, 10 μM pterostilbene and 30 μM allopurinol (n=6). Relative protein levels of Pitx2c were normalized to β-actin, respectively. Data are expressed as the mean ± S.E.M. $^{\#\#}P < 0.01$ *vs* normal cell control group; $^{*}P < 0.05$ *vs* fructose-vehicle cell group or fructose-vehicle + *p53* siRNA control cell group.

Figure S11. **Pterostilbene and allopurinol decrease TGF-β1-mediated CTGF expressions to inhibit downstream hypertrophic and fibrotic response-associated indicators ANP, α-SMA and FSP-1 expressions in fructose-exposed H9c2 cells.** Cellular protein levels of ANP (A), α-SMA (B) and FSP-1 (C) were determined in *CTGF* siRNA-transfected H9c2 cells co-incubated with 5 mM fructose, 10 μM pterostilbene and 30 μM allopurinol (n=6). Cellular protein levels of CTGF (D), ANP (E), α-SMA (F) and FSP-1 (G) were determined in *TGF-β1* siRNA-transfected H9c2 cells co-incubated with 5 mM fructose, 10 μM pterostilbene and 30 μM allopurinol (n=6). Relative protein levels of CTGF, ANP and FSP-1 were normalized to β-actin, of α-SMA were normalized to GAPDH, respectively. Data are expressed as the mean ± S.E.M. $^{\#}P < 0.05$, $^{\#\#}P < 0.01$ *vs* normal cell control group; $^{*}P < 0.05$, $^{**}P < 0.01$ *vs* fructose-vehicle cell group or fructose-vehicle + *CTGF* siRNA or

TGF-β1 siRNA control cell group.

Figure S12. Effects of pterostilbene and allopurinol on fructose-induced alteration of cellular CTGF, ANP, α-SMA and FSP-1 in NAC pretreated-, *Pitx2c* siRNA, miR-15b mimic, *p53* siRNA transfected-H9c2 cells. Cellular CTGF, ANP, α-SMA and FSP-1 protein levels were determined in NAC (A-D) pretreated-, *Pitx2c* siRNA (E-H), miR-15b mimic (I-L), *p53* siRNA (M-P)-transfected H9c2 cells co-incubated with 5 mM fructose, 10 μM pterostilbene and 30 μM allopurinol, respectively. Relative protein levels of CTGF, ANP and FSP-1 were normalized to β-actin, of α-SMA were normalized to GAPDH, respectively. Data are expressed as the mean ± S.E.M. $^{\#}P < 0.05$, $^{\#\#}P < 0.01$, $^{\#\#\#}P < 0.001$ *vs* normal cell control group; $^{*}P < 0.05$, $^{**}P < 0.01$, $^{***}P < 0.01$ *vs* fructose-vehicle cell group or fructose-vehicle + NAC or *Pitx2c* siRNA or miR-15b mimic or *p53* siRNA control cell group.

Figure S13. Proposed scheme of the mechanisms underlying fructose-induced myocardial fibrosis, as well as the attenuation of pterostilbene and allopurinol. High fructose triggers cardiac ROS to increase Pitx2c and then reduce miR-15b expression, this event up-regulates p-p53 to activate TGF-β1/Smads signaling in myocardial fibrosis. Pterostilbene and allopurinol with the reduction of ROS down-regulate Pitx2c to increase miR-15b expression, and then suppress p-p53-mediated TGF-β1/Smads signaling activation, resulting in the alleviation of fructose-induced myocardial fibrosis.

Supplementary Figures

Figure S1

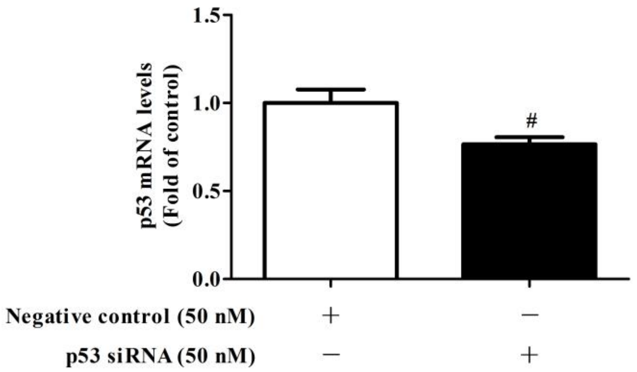

Figure S2

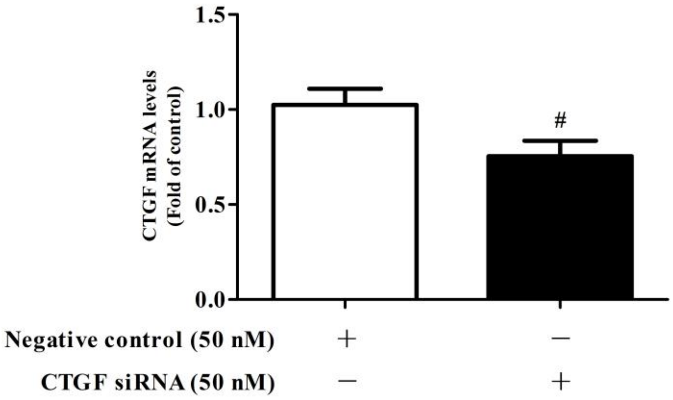

Figure S3

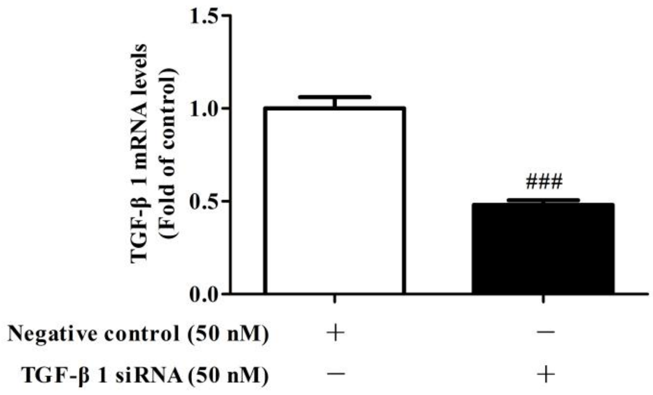

Figure S4

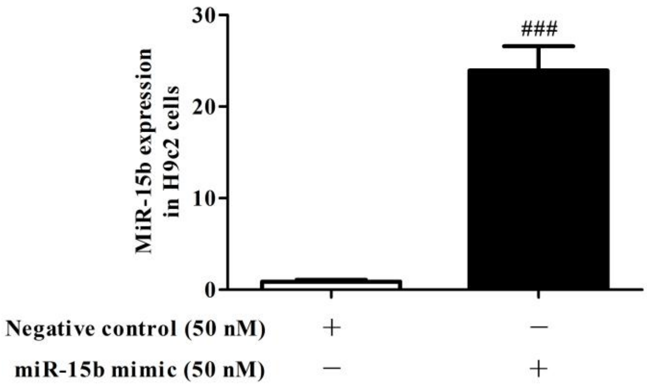

Figure S5

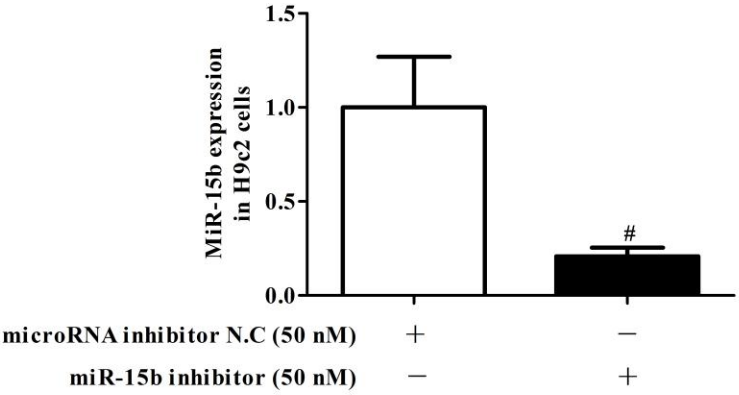

Figure S6

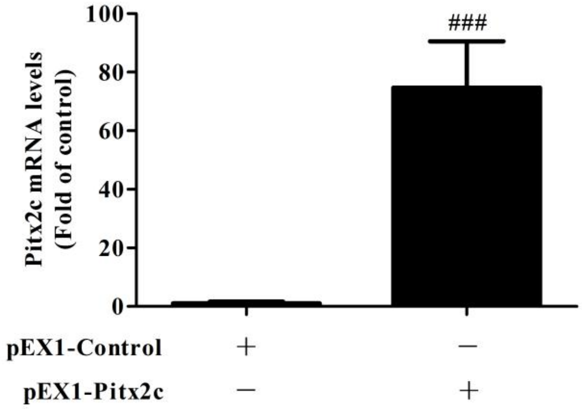

Figure S7

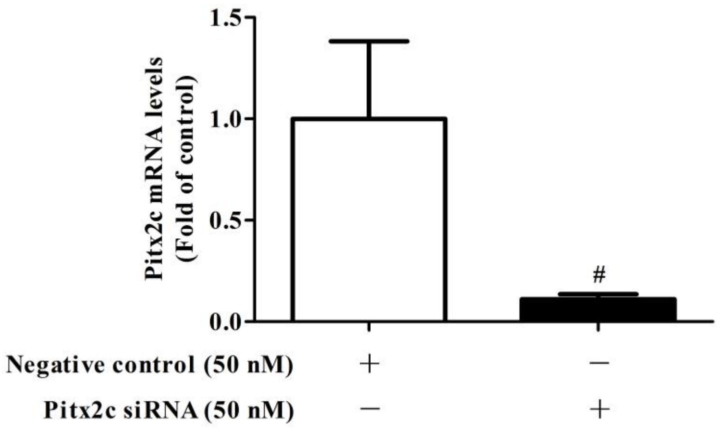

Figure S8

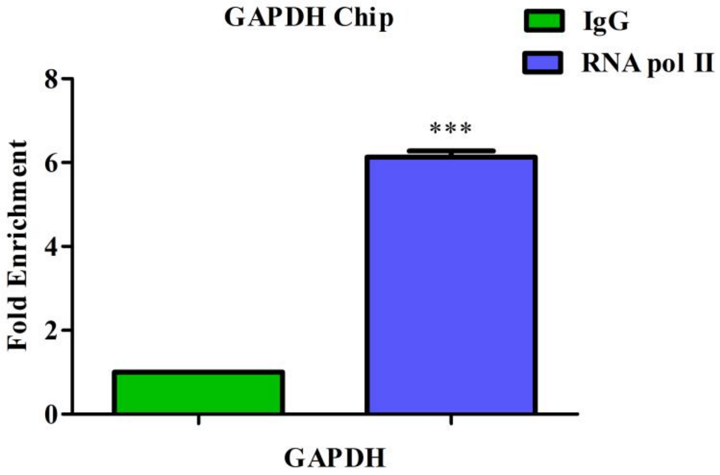

Figure S9

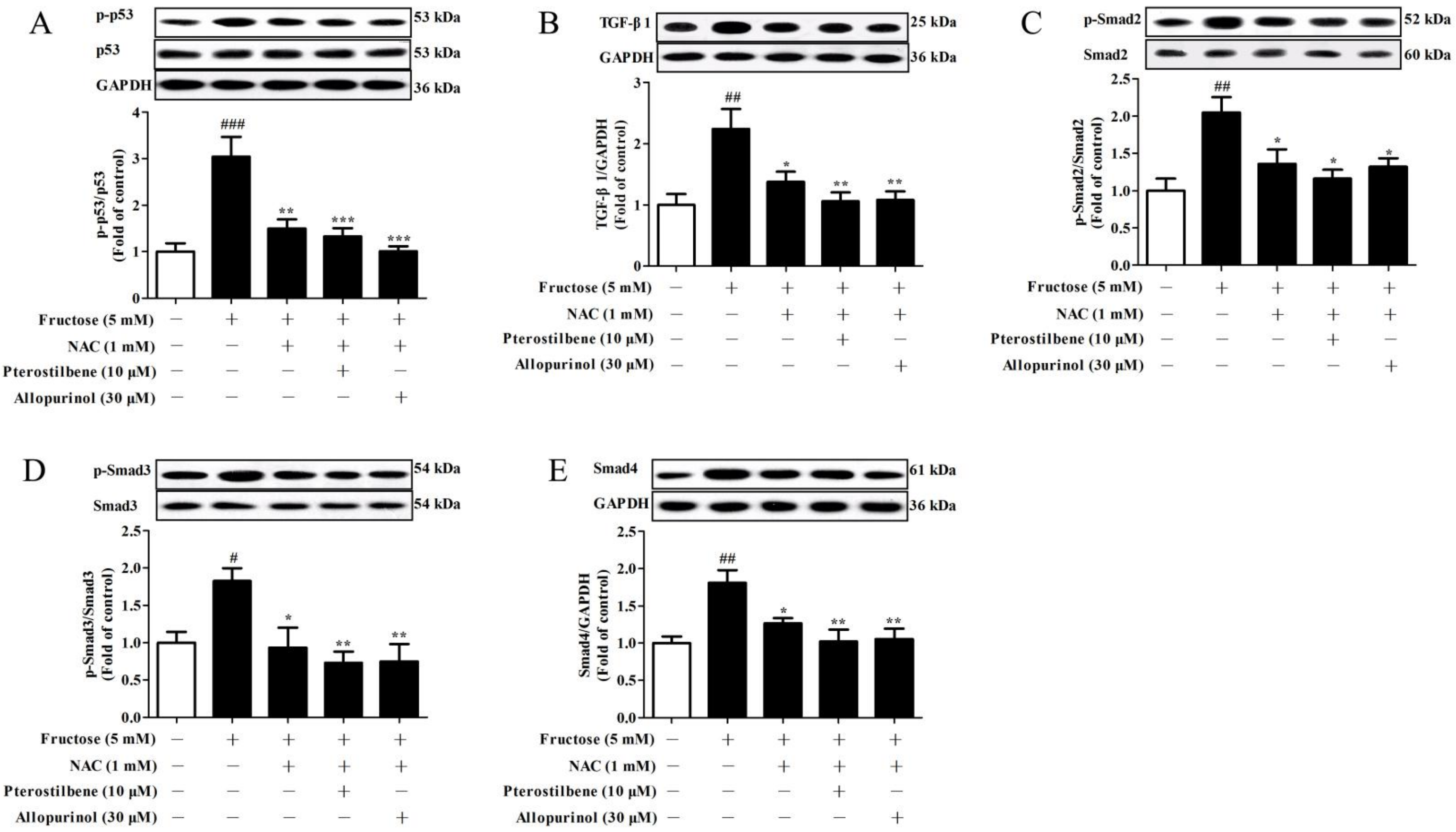
A
p-p53
53 kDa
p53
53 kDa
GAPDH
36 kDa
p-p53/p53 (Fold of control)
###
**
***
***
B
TGF-β 1
25 kDa
GAPDH
36 kDa
TGF-β 1/GAPDH (Fold of control)
##
*
**
**
C
p-Smad2
52 kDa
Smad2
60 kDa
p-Smad2/Smad2 (Fold of control)
##
*
*
*
D
p-Smad3
54 kDa
Smad3
54 kDa
p-Smad3/Smad3 (Fold of control)
#
*
**
**
E
Smad4
61 kDa
GAPDH
36 kDa
Smad4/GAPDH (Fold of control)
##
*
**
**
Fructose (5 mM)
NAC (1 mM)
Pterostilbene (10 μM)
Allopurinol (30 μM)

Figure S10

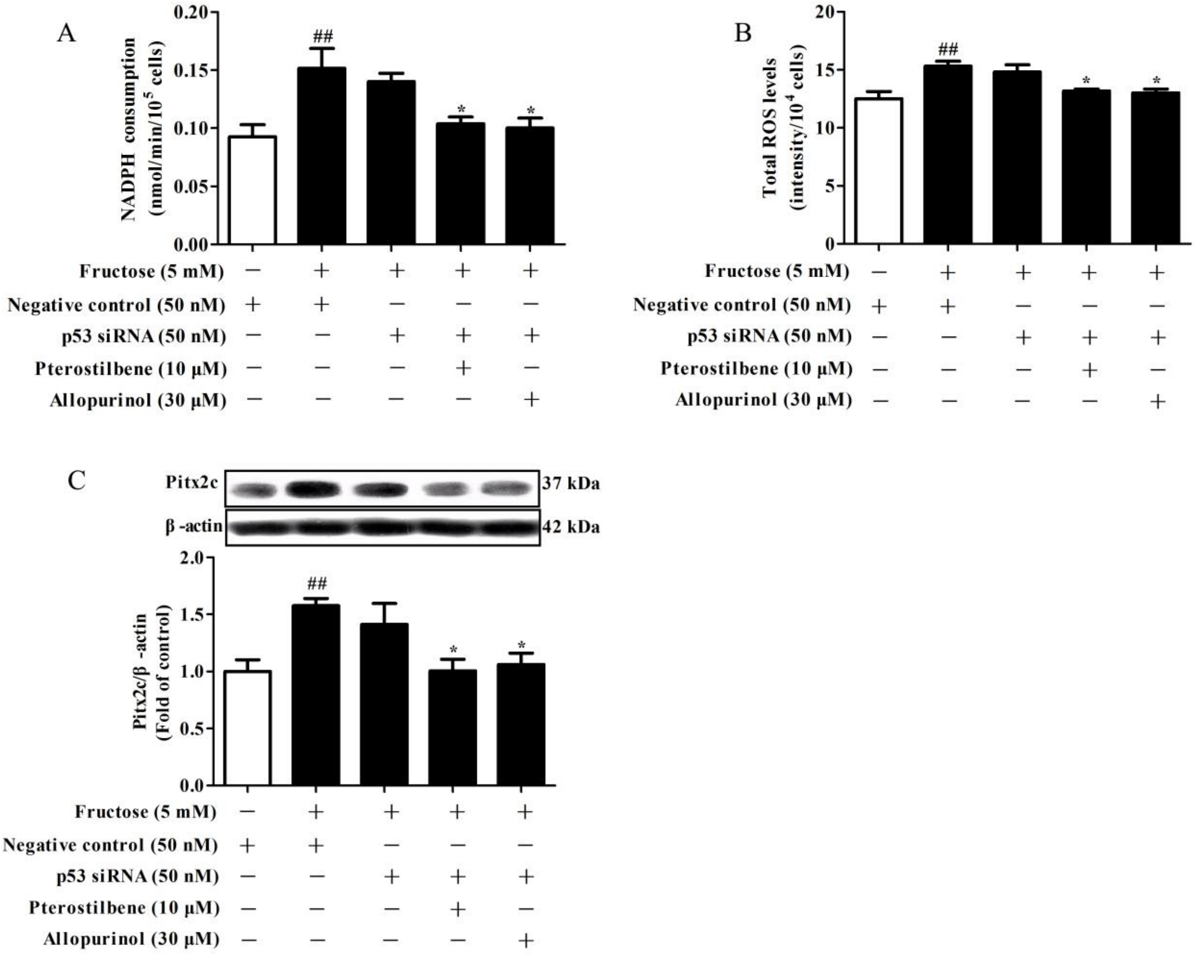

A
NADPH consumption (nmol/min/10^5 cells)
0.20
0.15
0.10
0.05
0.00
##
*
*
Fructose (5 mM) − + + + +
Negative control (50 nM) + + − − −
p53 siRNA (50 nM) − − + + +
Pterostilbene (10 μM) − − − + −
Allopurinol (30 μM) − − − − +
B
Total ROS levels (intensity/10^4 cells)
20
15
10
5
0
##
*
*
Fructose (5 mM) − + + + +
Negative control (50 nM) + + − − −
p53 siRNA (50 nM) − − + + +
Pterostilbene (10 μM) − − − + −
Allopurinol (30 μM) − − − − +
C
Pitx2c
37 kDa
β -actin
42 kDa
Pitx2c/β -actin (Fold of control)
2.0
1.5
1.0
0.5
0.0
##
*
*
Fructose (5 mM) − + + + +
Negative control (50 nM) + + − − −
p53 siRNA (50 nM) − − + + +
Pterostilbene (10 μM) − − − + −
Allopurinol (30 μM) − − − − +

Figure S11

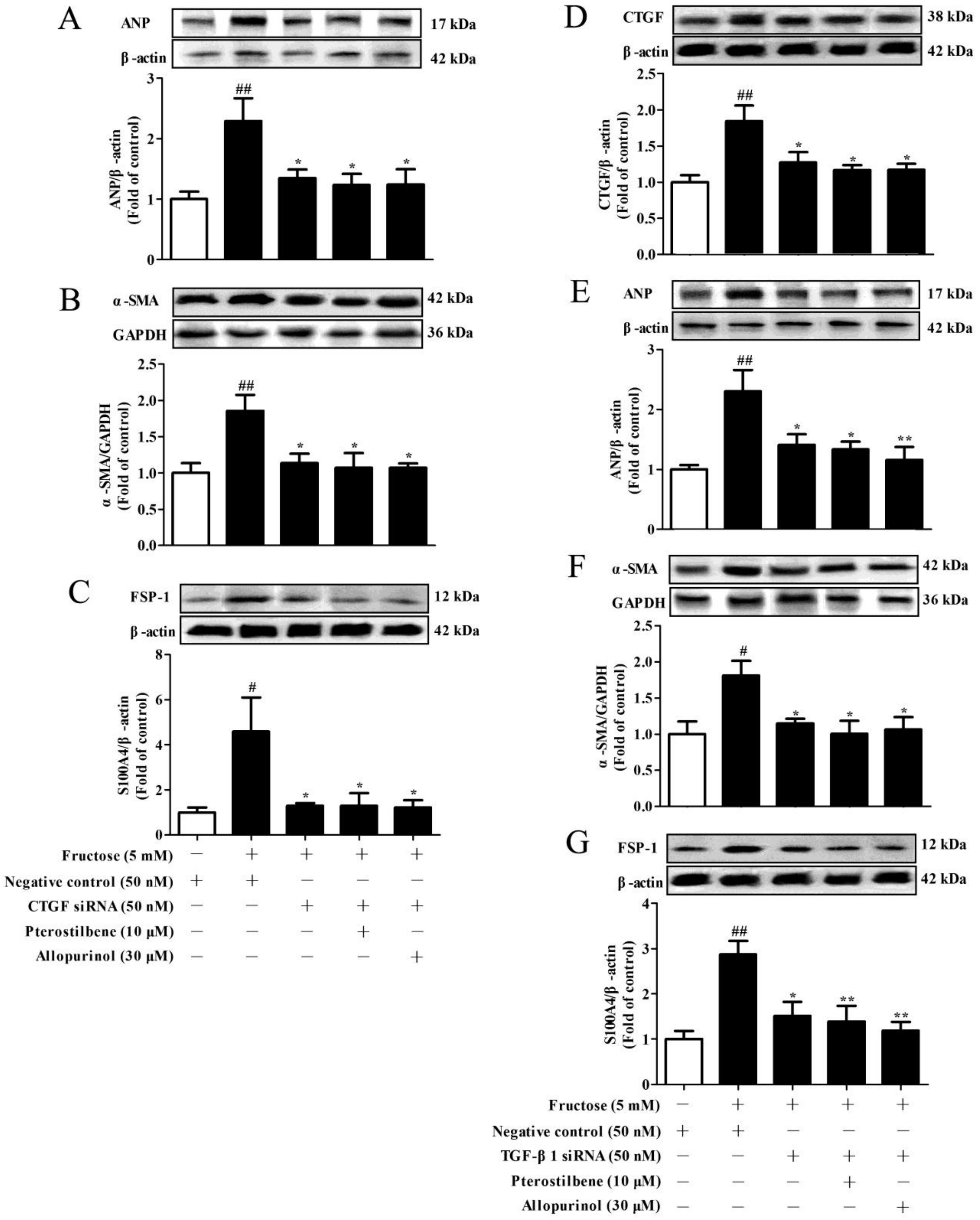
A
ANP
17 kDa
β -actin
42 kDa
ANP/β -actin (Fold of control)
B
α -SMA
42 kDa
GAPDH
36 kDa
α -SMA/GAPDH (Fold of control)
C
FSP-1
12 kDa
β -actin
42 kDa
S100A4/β -actin (Fold of control)
Fructose (5 mM)
Negative control (50 nM)
CTGF siRNA (50 nM)
Pterostilbene (10 μM)
Allopurinol (30 μM)
D
CTGF
38 kDa
β -actin
42 kDa
CTGF/β -actin (Fold of control)
E
ANP
17 kDa
β -actin
42 kDa
ANP/β -actin (Fold of control)
F
α -SMA
42 kDa
GAPDH
36 kDa
α -SMA/GAPDH (Fold of control)
G
FSP-1
12 kDa
β -actin
42 kDa
S100A4/β -actin (Fold of control)
Fructose (5 mM)
Negative control (50 nM)
TGF-β 1 siRNA (50 nM)
Pterostilbene (10 μM)
Allopurinol (30 μM)

Figure S12

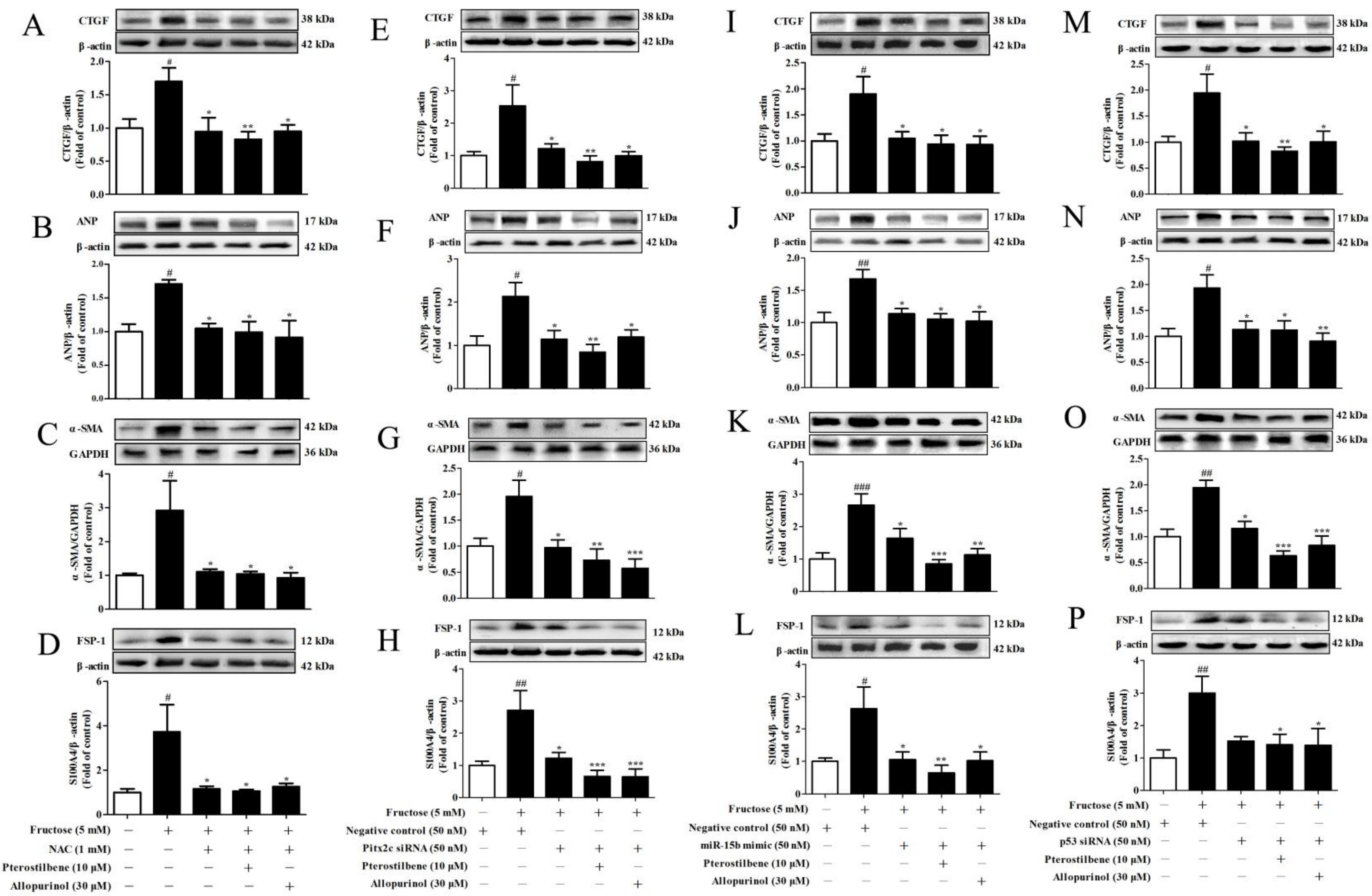

Figure S13

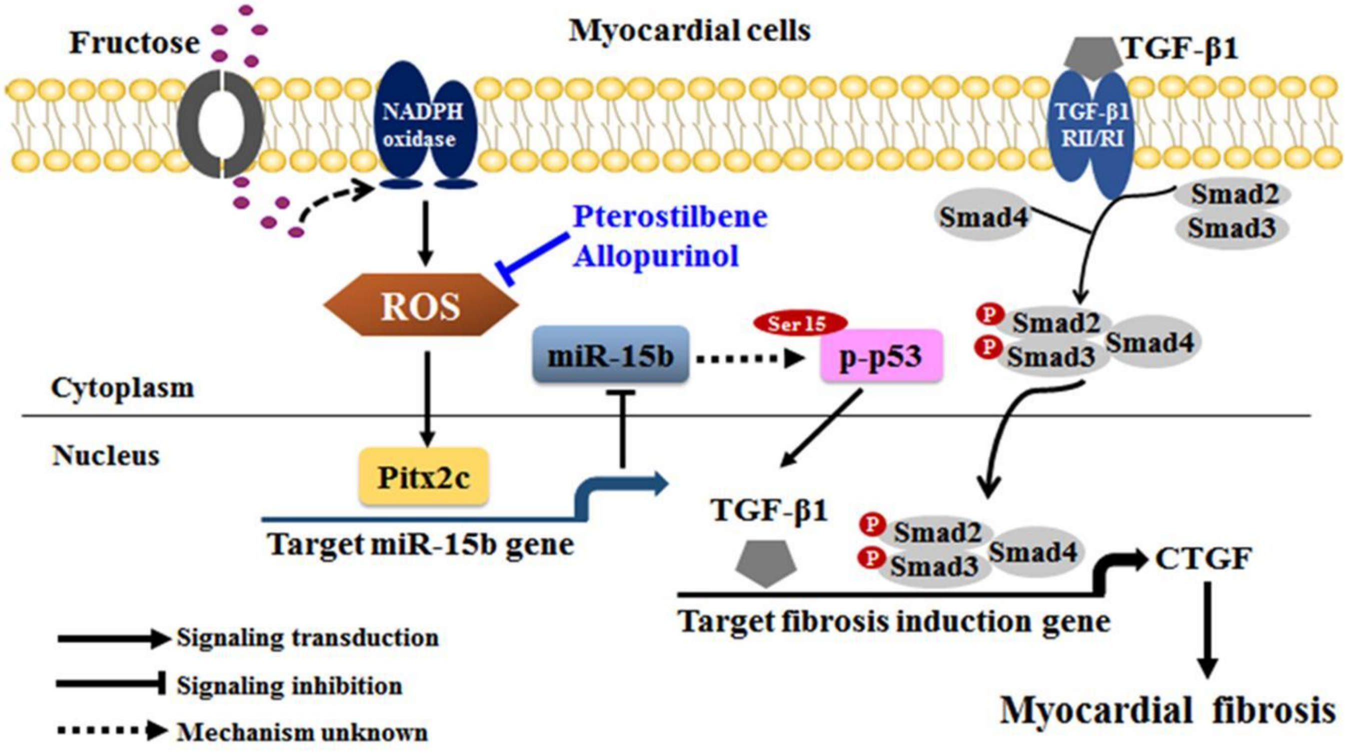
Fructose
Myocardial cells
TGF-β1
NADPH oxidase
TGF-β1 RII/RI
Smad4
Smad2
Smad3
Pterostilbene
Allopurinol
ROS
Ser 15
miR-15b
p-p53
P
Smad2
Smad3
Smad4
Cytoplasm
Nucleus
Pitx2c
Target miR-15b gene
TGF-β1
Smad2
Smad3
Smad4
CTGF
Target fibrosis induction gene
Signaling transduction
Signaling inhibition
Mechanism unknown
Myocardial fibrosis
